# Managing Pain in People with Cancer—a Systematic Review of the Attitudes and Knowledge of Professionals, Patients, Caregivers and Public

**DOI:** 10.1007/s13187-019-01548-9

**Published:** 2019-05-22

**Authors:** Salim M. Makhlouf, Simon Pini, Shenaz Ahmed, Michael I. Bennett

**Affiliations:** Academic Unit of Palliative Care, Leeds Institute of Health Sciences, School of Medicine, University of Leeds, Level 10 Worsley Building, Clarendon Way, Leeds, LS2 9NL UK

**Keywords:** Cancer pain management, Attitudes and knowledge, Professionals, Patients, Caregivers, Public, Systematic review

## Abstract

Cancer pain is a common symptom experienced by patients, caused either by the disease or its treatment. Morphine remains the most effective and recommended treatment for cancer pain. However, cancer patients still do not receive appropriate management for their pain, and under-treatment is common. Lack of knowledge and negative attitudes towards cancer pain and analgesia among professionals, patients and family caregivers are reported as one of the most common barriers to effective cancer pain management (CPM). To systematically review research on the nature and impact of attitudes and knowledge towards CPM, a systematic literature search of 6 databases (the Cochrane library, MEDLINE, PsycINFO, CINAHL, Web of Science and EMBASE) was undertaken in July 2018. Additionally, hand-searching of Google, Google Scholar and reference lists was conducted. The inclusion criteria were adult (18–65 years of age), studies which included attitudes and knowledge towards CPM, studies written in English, published literature only and cross-sectional design. Included studies were critically appraised by two researchers independently using the Joanna Briggs Institute Analytical Cross Sectional Studies Assessment (JBI-ACSSA). A total of 36 studies met the inclusion criteria. The main finding was that among professionals, patients, caregivers and the public there were similar attitudinal barriers to effective CPM. The most commonly cited barriers were fear of drug addiction, tolerance of medication and side effects of opioids. We also found differences between professional groups (physicians versus nurses) and between different countries based on their potential exposure to palliative care training and services. There are still barriers to effective CPM, which might result in unrelieved cancer pain. Therefore, more educational programmes and training for professionals on CPM are needed. Furthermore, patients, caregivers, and the public need more general awareness and adequate level of knowledge about CPM.

**Electronic supplementary material:**

The online version of this article (10.1007/s13187-019-01548-9) contains supplementary material, which is available to authorized users.

## Introduction

Cancer has become the most common cause of death worldwide [7, 86]. It has been estimated that by 2030, there will be about 21.4 million new cancer cases annually, and approximately 13.3 million cancer patients expected to be die from the disease [31]. Pain related to cancer is a common problem that can occur among patients who are having active cancer treatment [47].This can be a result of some complications following treatment of cancer, which can be physical or psychological symptoms [21, 73]. The prevalence of cancer pain can be associated with the stage of disease and the location of cancer [36, 41]. According to a recent meta-analysis, pain was reported by more than 50% of cancer patients who received anti-cancer treatment and about 66% of patients with advanced and metastatic cancer [26]. Several attempts have been made to establish effective CPM. One of the most important attempts is the “analgesic ladder,” established by the World Health Organisation (WHO), to manage cancer pain in adult patients [91]. Morphine remains the most effective and recommended treatment for CPM [99, 103]. Despite the improving quality of pharmacological options for pain management, several studies have revealed that patients at different stages of their disease still do not receive appropriate CPM [3, 18, 25, 37, 52, 92]. Lack of knowledge and negative attitudes towards CPM among professionals [1, 18, 80, 82, 93], cancer patients [61] and family caregivers [79] were reported by recent reviews and studies as one of the most common barriers to effective CPM.

Numerous studies conducted worldwide have assessed independently either professionals’, patients’, caregivers’, or the publics’ attitudes and knowledge towards CPM. However, synthesis of these results has not yet been undertaken. Conducting such a review is important as it is now well established from a variety of studies that many common barriers delay the delivery of effective CPM to patients; this could be caused by professionals [9, 11, 18, 24, 46, 80, 83, 84], cancer patients [57], caregivers [95] and the general public [51], which is likely to result in inadequate CPM. Thus, the aim of this systematic review is to determine the nature and impact of attitudes and knowledge towards CPM.

## Methods

### Protocol and Registration

The preferred reporting items for systematic reviews and meta-analysis (PRISMA) statement has been used as a guideline for reporting the findings in this systematic review [53, 63, 85]. The protocol for this review was registered with PROSPERO; the registration number is CRD42018117625.

### Adapting PICO into PCO for This Current Systematic Review

The types of studies, participants and interventions, as well as the types of outcome measures (PICO) will be modified to PCO (population, context and outcome) as there are no interventions or comparisons needed. [78, 89]. For more details, see Table 1.

**Table 1:** Example of systematic review: PICO modified to PCO (population, context and outcome)

Population	Professionals, adult cancer patients, family caregivers of patients with cancer and general public aged 18 to 65) years old
Context	Caner pain and opioids
Outcome	Attitudes and knowledge

Adapted from Butler et al. [15]

**Table 2:** Summary of inclusion and exclusion criteria

Inclusion criteria	Exclusion criteria
• Adult (18–65 years of age) • Studies written in English • Cancer pain • Studies include attitudes and knowledge towards cancer pain and opioid • Published literature only • Cross-sectional design	• Children and adolescents (< 18 years of age) • Studies not in English • Pain related to non-malignant disease • Barriers not related to attitudes and knowledge • Unpublished research

### Eligibility Criteria: Population, Context and Outcome

The inclusion and exclusion criteria are listed in Table 2

### Search Strategy for Identification of Studies

In this systematic review, we searched 6 electronic databases (the Cochrane library, MEDLINE, PsycINFO, CINAHL, Web of Science and EMBASE) in July 2018. Additionally, hand-searching of Google, Google Scholar and reference lists was conducted. The search terms were based on population, contexts (context pain, context opioids and context cancer) and outcome [16]. To identify publications for inclusion in the present systematic review, the keywords employed were as shown in Table 3. For more information regarding search strategy, see Appendix 5.

### Data Extraction

The data extraction form was developed and piloted independently by two reviewers (SM & SP). A third reviewer (MB) was involved to reconcile any disagreements. Using data extraction forms can potentially reduce bias and improve validity and reliability [17]. In this review, the data extraction form was adapted from Centre for Reviews and Dissemination, University of York [17] (see Appendix 1). The extraction of data from the included studies was based on the names of authors, year, country of publication, design of study, the aim of study, sample size, the setting of study, mean age, sex ratio, type of measurements, type of sample, type of cancer, main findings and the quality of study as outlined in Table 4.

### Quality Assessment of the Included Studies

The reason for using a critical appraisal process for the included studies was that studies can be published with variable levels of methodological rigour and therefore their results could be unreliable [15]. It has been strongly recommended that the assessment of quality should be done separately by at least two reviewers [56, 64, 72, 90]. Accordingly, all 36 included studies have been critically apprised by two researchers (SM & SP) independently using the Joanna Briggs Institute Analytical Cross Sectional Studies Assessment (JBI-ACSSA) (see Appendix 2). To reconcile any differences, a third reviewer (MB) was involved. The JBI-ACSSA tool was chosen as it is appropriate for the study design of included quantitative studies [64, 90]. The assigning score for the quality of the data was performed as 1 point for each applicable item with a score of 7 as the maximum score [74]. An overall score was calculated for each included study and the rating of quality was judged as good (6/7 and 7/7), fair (3/7 to 5/7) or poor (< 3/7) [35] (see Appendix 3). No score was below 3/7, so no study was excluded based on the quality assessment only.

## Results

### Information Sources and Study Selection

**Fig. 1:**
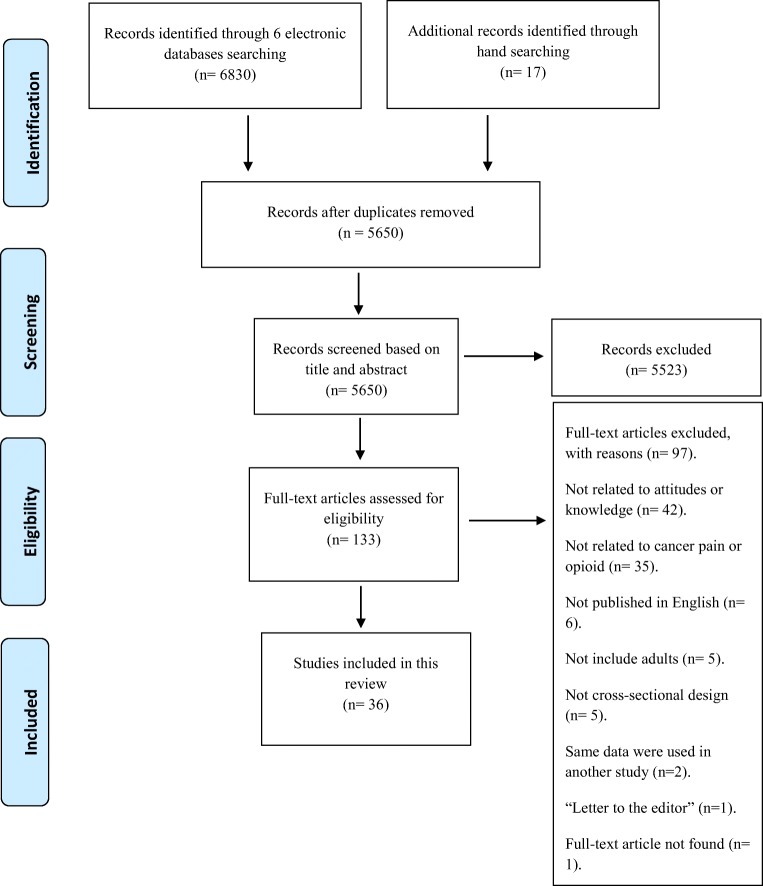
PRISMA diagram for strategy of the study selection. Adapted from Moher et al. [62]

The total number of studies identified by 6 electronic databases (the Cochrane library, MEDLINE, PsycINFO, CINAHL, Web of Science and EMBASE) was 6830 articles (see Appendix 5). In addition, 17 studies were identified by hand-searching (including Google, Google Scholar and checking the reference lists). Among these 6847 studies, 5650 articles were included after the duplicate studies were removed. Among the 5650 included studies, 5523 studies were excluded after the title and abstract of each study were carefully reviewed. The total number of full-text articles assessed for eligibility was 133. A further 97 studies were excluded and all full references of these excluded articles and the reasons for exclusion are listed in Appendix 4. Consequently, a total number of 36 studies were included in this review as illustrated in Fig. 1.

**Table 3:** Example of PCO search terms

Population	Context pain	Context opioids	Context cancer	Outcome
adults*	exp PAIN/	exp Analgesics/	Cancer*	Attitude*
	exp Pain management/	exp morphine/	tumor*	Knowledge*
	pain*	exp narcotics/	carcinoma*	View*
	Management* management*,	morphine*	leuk?emia*	opinion*
		Buprenorphine*	metasta*	concern*
		codeine*	malignan*	belief*
		opium*	lymphoma*	feeling*
		diamorphine*	melanoma*	idea*
		opioid*	oncolog*	perception*
		Dihydrocodeine*	exp neoplasms/	perspective*
		opiate*		experience*
		alfentanil*		perceive*
		fentanyl*		standpoint*
		oxycodone*		expectation*
		hydromorphone*		preference*
				need*
				satisfaction*
				interaction*

Adapted from Butler et al. [15]

### Characteristics of Included Studies

The 36 studies included in this review used a cross-sectional design, employing various questionnaires, to assess knowledge of and attitudes towards CPM. The studies were based in 18 countries. The characteristics of included studies are illustrated in Table 4.

**Table 4:** The characteristics of 36 included studies

Author (s), year, and country	Study design	Study aim	Sample size	Study setting	Mean age	Sex ratio	Measurements	Type of sample	Type of cancer	Main finding	Quality scoring
Breuer et al., (2011), New York, USA	Cross-sectional method	To evaluate the attitudes, knowledge and practices of US medical oncologists that are related to management of cancer pain	482 oncologists	The American Medical Association’s Physician Master File	56 years (range 51–61 years).	Male 80%, female 20%	Not stated	US medical oncologists	N/A	The most important barriers to CPM were poor assessment (median, 6; IQR, 4 to 7) and patient reluctance to take opioids (median, 6; IQR, 5 to 7) or report pain (median, 6; IQR, 4 to 7). Other barriers included physician reluctance to prescribe opioids (median, 5; IQR, 3 to 7) and perceived excessive regulation (median, 4; IQR, 2 to 7). In response to two vignettes describing challenging clinical scenarios, 60% and 87%, respectively, endorsed treatment decisions that would be considered unacceptable by pain specialists. Frequent referrals to pain or palliative care specialists were reported by only 14% and 16%, respectively.	6/7
Bernardi et al., (2007), Italy	Cross-sectional method	To obtain information about the knowledge and attitudes of Italian oncology nurses concerning CPM and to determine the predictors of nurses’ PM knowledge	287 nurses	Oncology wards in the north, centre and south of Italy	35 (22–56)	Male (19.2%), female (78.7%)	the Nurses’ Knowledge and Attitudes Survey Regarding (NKARSP)	Oncology nurses	N/A	Among the 39 questions examined, the mean score for correctly answered items was 21.4 (55% correct answer). Among the 39 items surveyed, 23 received less than 60% of the correct answer rate. Further analysis of items showed that more than 50% of oncology nurses underestimated the pain of patients and they did not treat it in a correct way and they had an incorrect self-evaluation about their PM knowledge. 90.2% of respondents did not know the correct percentage of patients who over report their pain.	6/7
Colak et al., (2014), Turkey	Cross-sectional method	To survey the attitudes of cancer patients towards morphine use for CPM in a MMC and identify the factors influence patient decisions to accept or refuse morphine for CPM	488 cancer patients with pain	Three different Education and Training Hospitals (ETH) located in 3 cities of Central Anatolia: Ankara, Konya and Kayseri; namely Diskapi Yildirim Beyazit ETH, Kayseri ETH and Konya	54 (range: 18–87) years	Female 301, male 187	Not stated	Patient with cancer	Breast 217, colorectal 97, gastric 63 and lung 37 patients	About 50% of cancer patients refused to use morphine and 36.8% of them prefer another drug due to fear of addiction. Reservation of morphine for later in their disease was the case for 22.4% of the patients who refused morphine use. Whereas, 13.7% of cancer patients reused morphine and 9.7% of them preferred another medication as a result of religious reasons. Both before and after the description only 12% of the patient reported they would not use morphine even if it was recommended.	6/7
Cohen et al., (2005), Israel	Descriptive cross-sectional method	To explore cancer pain experience, including knowledge and attitudes towards pain and pain control	39 cancer patients with pain	Radiation department and outpatient centre of a large academic medical institution in Israel	73.2 (range 65–88; SD = 5.4) years	Male (48.7), female (51.3)	Patient Pain Questionnaire Knowledge Subscale (PPQK)	Cancer patient	Lung 12%, breast 33.3%, colon 7.7%, other 30.8%	Over half (56.7%) reported severe worst pain and had negative pain management indexes (56.4%). knowledge and attitudes towards pain and pain control were poor (54.55%).	7/7
Darawad et al., (2017), Jordan	Descriptive cross-sectional method	To compare physicians’ and nurses’ knowledge and attitudes towards cancer pain management (CPM) and describe their perceived barriers to CPM at cancer units	207 participants (72 physicians and 135 nurses)	Oncology units from the military, educational, oncology centre and public sectors in Jordan	Nurses:28.1, physicians 30.5	Nurses (M 54.8%; F 45.2%); physicians (M 61.1%; F 38.9%)	The Knowledge and Attitudes Survey Regarding Pain (KAS)	72 physicians and135 nurses	N/A	Findings revealed that both physicians and nurses had fair knowledge and attitudes towards CPM. Physicians had significantly higher knowledge and better attitudes than nurses (62.3% vs. 51.5%, respectively). Physicians were knowledgeable about medication for PM and opioid addiction but had negative attitudes towards CPM. Nurses’ knowledge was better in regard of CPM guidelines, while they had poor knowledge about pharmacological PM and opioid addiction. Physicians and nurses perceived knowledge deficit, lack of PM, opioid unavailability and lack of psychological interventions as the most common barriers to CPM.	5/7
Eftekhar et al., (2007), Iran	Cross-sectional method	To evaluate knowledge about and attitudes towards cancer pain and its management in Iranian physicians with patient care responsibilities	55 physicians in six university hospitals	Physicians (haematologists, oncologists, surgeons, internists, gynaecologists, radiotherapists) in six university hospitals in Tehran	37 (ranged 28–65) years.	54.6% male responders	Not stated	Physicians	N/A	Physicians recognised the importance of PM priority (76%) and about one half of the physicians acknowledged the problem of inadequate PM in their settings. Inadequate staff knowledge of PM as barriers to good PM. No correlation was found between what physicians think they know and what they know about cancer pain and its management.	4/7
Elliott et al., (1996), USA	Cross-sectional method	The study reported here investigated the relationship between specific knowledge and attitudes (cognitive factors) and patients’ and family members’ reports of pain due to cancer	244 participants, 122 cancer patients and 122 family members	MCPP communities, medical service areas	64 years for cancer patients and 60 years for family members	Cancer patients 53% female; family members 62% female	Not stated	Cancer patients and family member	N/A	Patients’ and their families’ reports of patient pain and performance status were highly correlated, although family members consistently reported more pain and disability. Using regression analysis, cognitive factors were strongly related to family reports of patients’ pain (*R*^2^ = 0.27), but contributed little to explaining pain reported by patients themselves (*R*^2^ = 0.06). Improved understanding of patients’ pain assessments depends on further investigation of other cognitive factors and of sensory and affective factors. Assessment of pain for family members are significantly related to appropriate knowledge and attitudes.	6/7
Elliott et al., (1995), USA	Cross-sectional method	To determine knowledge and attitudes about CPM among physicians in six Minnesota communities and to determine the physician-related barriers to optimal CPM	145 physicians	The Minnesota Cancer Pain Project (MCPP)	Not stated	Male 89.7%, female 10.3%	Cross-sectional telephone survey, the physician survey instrument	Physicians	N/A	Significant knowledge deficits were identified in nine of 14 CPM principles, but inappropriate attitudes were found in only two of nine CPM concepts. Medical specially had the strongest influence on knowledge and attitudes, with primary care physicians having significantly better outcomes than surgeons or medical subspecialists.	7/7
Elliott and Elliott, (1992), State of Minnesota, USA	Cross-sectional method	To explore the prevalence among practicing physicians of 12 proposed myths or misconceptions about the use of morphine in CPM	150 physicians	Direct patient care in Duluth, Minnesota. 47 different medical schools located in 31 states, Canada, and England.	Older MDs, *N* = 41, middle-aged MDs *N* = 53,younger MDs *N* = 56	It is not stated.	Physician Cancer Pain Attitude Questionnaire	Physicians	N/A	Many physicians misunderstood concepts of morphine tolerance, both to analgesia (51%) and to side effects (39%). Many were unaware of the use of adjuvant analgesics (29%), efficacy of oral morphine (27%) and non-existent risk of addiction in CPM (20%).	7/7
Furstenberg et al., (1998), State of New Hampshire, USA	Cross-sectional method	Evaluate the knowledge and attitudes of all three types of providers directly involved in caring for CPs and identify areas where deficiencies exist in order to target future educational efforts appropriately	554 participants: 188 physicians, 118 pharmacists and 248 nurses.	Research and Development Committee of the New Hampshire State Cancer Pain Initiative based on a review of questionnaires used in similar studies.	43.4 years	Male 44%, female 56%	Not stated	Physicians, pharmacists, nurses	N/A	The results are generally consistent with results from other studies of physicians, nurses and pharmacists in terms of knowledge of and attitudes towards CPM, perceived barriers to effective CPM and lack of training in CPM. In contrast to some earlier studies, however, providers in this sample were not concerned about addiction among CPs. Knowledge deficits were found across providers. This negative finding is consistent with data from a number of recent studies and suggests that some progress has been made in allaying provider concerns in this area.	6/7
Gallagher et al., (2004), British Columbia	Survey	To acquire current data on physician knowledge and attitudes towards CPM as an educational needs assessment for the UBC Division of Palliative Care. Also to solicit physicians’ opinions about the TPP’s possible effect on CP prescribing	4618 physicians	Palliative care at the University of British Columbia, the BC Cancer Agency and the College of Physicians /Surgeons of BC.	Not stated	Male (67.9%), female (27.9%)	Not stated	British Columbia physicians	N/A	The results show 12.0% of MDs agreed at knowledge question that any Pt given opioids for CPM is at a 25% or more risk for addiction. The highest percent of 80.6% disagreed that morphine for CPM shortens life but makes people more comfortable. The questions most frequently answered incorrectly (or by “do not know”) were those about equi-analgesic dosing (68%) and adequate breakthrough dosing (45%), revealing knowledge deficiencies that would significantly impair a physician’s ability to manage CP. The result shows that there were high scores in the attitude questions but larger deficits in knowledge about CPM.	6/7
Ger et al., (2000), Taiwan	Cross-sectional method	To examine the attitudes of MDs regarding the optimal use of analgesics for CPM, to evaluate their knowledge and attitudes towards opioid prescribing and to comprehend their perceptions of the barriers to optimal CPM	204 physicians with cancer patient care	Two medical centres, Kaohsiung Veterans General Hospital (KSVGH) and Tri-Service General Hospital (TSGH), in Taiwan	36.4 years	Males (95%) and females (5%)	Not stated	Physicians	N/A	The most important barriers to optimal CPM identified by physicians themselves were physician-related problems, such as inadequate guidance from a pain specialist, inadequate knowledge of CPM and inadequate pain assessment. The results of his study suggest that active analgesic education programmes are urgently needed in Taiwan.	6/7
Hollen et al., (2000), South Central State, USA	Cross-sectional method	To identify knowledge strengths and weaknesses and misperceptions about CPM between two groups of reg nurses in different setting	64 hospice and hospital oncology unit nurses	7 adult hospital oncology units and 11 hospices in a South Central State	45 (10.54) for hospice nurses and 40 (9.32) for hospital nurses	It is not stated.	North Carolina Cancer Pain Initiative (NCCPI) survey	Hospice (*n* = 30) and hospital (*n* = 34) nurses.	N/A	Hospice nurses (*X* = 24.71, SD = 2.27) scored significantly higher on the total knowledge test than the hospital oncology nurses (*X* = 20.76, SD = 3.77; *t* [61] = 5.09, *p* = 0.0001). Hospice nurses also scored significantly higher than hospital nurses on opioid subscale (*t* [62] = 5.52, *p* = 0.0001) and scheduling subscale (*t* [63] = 3.77, *p* = 0.0004). Regarding attitudes, hospice nurses also had significantly higher liberalness score (*X* = 18.31, SD = 1.79) than hospital nurses (*X* = 16.94, SD = 2.32; *t* [62] = 2.58, *p* = 0.0122).	5/7
Jho et al., (2014), Korea	Cross-sectional method	To evaluate knowledge, practices and perceived barriers regarding CPM among physicians and nurses in Korea	333 physicians and nurses	11 hospitals (6 public and 5 private hospitals) across Korea	33.2 years for physicians and 29.0 years for nurses	Physician, 61.5% male, 38.5% female. nurses, 0% for male and 100% female	Not stated	Physicians (*n* = 149) and nurses (*n* = 284).	N/A	Nurses performed pain assessment and documentation more regularly than physicians did. Although physicians had better knowledge of PM than did nurses, both groups lacked knowledge regarding the side effects and pharmacology of opioids. Physicians working in the palliative care ward and nurses who had received PM education obtained higher scores on knowledge. Physicians perceived patients’ reluctance to take opioids as a barrier to pain control, more so than did nurses, while nurses perceived patients’ tendency to under-report of pain as a barrier, more so than did.	6/7
Jeon et al., (2007), Korea	Cross-sectional method	To assess clinicians’ practices and attitudes about CPM and to identify perceived concerns about and barriers to pain control in urban cancer-treatment settings in Korea	250 physicians and nurses	7 hospitals in Korea	Not stated	Male 107 (42.8%), female 143 (57.2)	Not stated	Physicians and nurses	N/A	The result shows that both groups identified 90.6% concerned that difficulty in controlling strong side effects as the biggest potential barrier to good pain control. Also, they identified inadequate assessment of pain and pain management with 78.5% as the second biggest potential barrier to good pain control. 64.5% of both groups stated inadequate staff knowledge of PM.	6/7
Kassa and Kassa, (2014), Ethiopia	Cross-sectional method	To assess the attitude, practice of nurses’ and barriers regarding CPM at selected health institutions offering cancer treatment in Addis Ababa city,Ethiopia, 2013	82 nurses	1 public and 4 private health institutions that provide cancer treatment in Addis Ababa, the capital city of Ethiopia	42 years	Male 18 (22%), female 64 (78%).	Nurses’ Knowledge and Attitudes Survey Regarding Pain (NKARSP)	Nurses	N/A	More than half, 53.7%, of the nurses have a negative attitude towards CPM. Similarly 65.9% of nurses’ had poor CPM practice. Lack of courses related to pain in the under graduate classes, lack of continuing training, patient and work overload, role confusion, lack of motivation including salary were the identified barriers for adequate pain management. Monthly income of greater than 1500 Ethiopian Birr (ETB) were found to be associated with attitude towards cancer pain management (CPM) (AOR = 0.16, 95% CI = 0.03–0.78).	6/7
Kaki, (2011), Saudi Arabia	Cross-sectional method	To assess the final year medical students’ knowledge, beliefs and attitude towards cancer pain, and the need for a formal pain curriculum in medical schools	325 the sixth year medical students	King Abdul-Aziz University Hospital, Jeddah, Kingdom of Saudi Arabia	23 years (42.9%)	Males (*n* = 158) and females (*n* = 167)	Not stated	Sixth year medical students	N/A	54% of the respondents believed that < 40% of CPs suffered from pain. 46% of them considered CP untreatable, while 41.6% considered pain a minor problem and 58.6% considered the risk of addiction is high with legitimate opioids’ prescription. There are 23.1% of students believed that patients are poor judges of their pain, 68% of them limited opioids prescription to patients with poor prognosis and 77.1% believed that drug tolerance or psychological dependence, rather than advanced stages’ cancer is the cause of increasing analgesic doses. The students’ knowledge on the causes of CP, pain clinic rule and pain inclusion in the medical curriculum was poor.	4/7
Kim et al., (2011), South Korea	Cross-sectional method	To evaluate young Korean physicians’ attitude towards the usage of analgesics for CPM and their optimal knowledge of opioid prescription Also wanted to find out the real factors that affect the attitude and knowledge of doctors.	1204 physicians	National Cancer Centre, Goyang-Si, Gyeonggi-do, South Korea	29.9± 2.2 years	Male 100%	Not stated	Internal medicine and family medicine doctors, surgeons, anaesthesiologists, paediatricians and general physicians	Gastric, lung, liver and colorectal malignancies for males and gastric, breast, colon, rectum, uterine cervix, lung and thyroid gland malignancies for females	A large sample of physicians showed a negative attitude and inadequate knowledge status about CPM. The degree of attitude and knowledge status was different as their specialties and personal experiences. The factors that affected doctors’ attitude and knowledge were: (1) medical specialty, (2) past history of using practical pain assessment tool, (3) self-perception of knowledge status about PM, (4) experience of prescribing opioids, and (5) experience of education for CPM. Although many physicians had a passive attitude in prescribing analgesics, they are willingly open to use opioids for CPM in the future. The most important perceived barriers to optimal CPM were the fear for risk of tolerance, drug addiction, side effects of opioids and knowledge deficit about opioid.	7/7
Lou and Shang, (2017), China	Descriptive cross-sectional method	To investigate patients’ attitudes towards cancer pain management and analyse the factors influencing these attitudes	726 cancer patients and their caregivers	The oncology department of 7 hospitals in Beijing, China	Patients: 54.39± 12.72 (range, 18–88) years, caregivers 46.07 ± 13.26 (range, 18–76) years	Patients: male 52.34%, female 47.66%, caregivers, male 45.73%; female 54.27	Pain Management Barriers Questionnaire-Taiwan Form (BQT), and Pain Knowledge Questionnaire	Cancer patients (*n* = 363) and their caregivers (*n* = 363)	Lung, oral, nasopharyngeal, oesophageal, gastrointestinal, breast, liver, pancreatic, lymphoma, kidney, ureter, bladder, ovarian, and uterine	The average score of attitudes towards CPM for CPs and caregivers through the BQT subscale score ranged from 0 to 5 were 2.96 ± 0.49 and 3.03 ± 0.49, respectively. The dimension scores for CPs and CGs indicated good attitudes in three areas (scores < 2.5), “desire to be good” (2.22), (2.38), “fatalism” (2.08), (2.31) and “religious fatalism” (1.86), (2.02), and poor attitudes in six areas (scores ≥ 2.5), “tolerance” (3.83), (3.74), “use of analgesics as needed (p.r.n.)” (3.73), (3.51), “addiction” (3.44), (3.43), “disease progression” (3.28), (3.27), “distraction of physicians” (3.16), (3.01), and “side effects” (2.99), (3.22). Two factors were entered into the regression equation: the caregivers’ attitudes towards CPM and the patients’ pain knowledge. These two factors explained 23.2% of the total variance in the patients’ average scores for their attitudes towards CPM.	7/7
Larue et al., (1999), France	Cross-sectional method, mixed method	To assess the evolution of the knowledge and attitudes of the French population with respect to pain management and morphine use	2007 general population: 1001 general population in 1990 and 1006 general population in 1996	Telephone surveys by professional interviewers, and structured questionnaires	35–44 years, 168/1001 (17%) in 1990 and 201/1006 (20%) in 1996	Male 470/1001 (47%) in 1990 and 474/1006 (47%) in 1996.	Not stated	General population in France	Not stated	The respondents’ awareness of the occurrence of pain in the course of cancer improved: 65% (656 of 1006) thought that pain is rare at early stages of cancer in 1996, compared with 49% (490 of 1001) in 1990; 84% (845 of 1006) thought that pain is frequent at advanced stages of cancer, compared with 72% (724 of 1001) in 1990. Proportion of people who were not afraid of becoming addicted to morphine if prescribed for pain relief increased from 26% (263 of 1001) in 1990 to 69% (699 of 1006) in 1996. However, the proportion of respondents who agreed that morphine can be prescribed to CPs increased only slightly, from 79% (790 of 1001) to 83% (833 of 1006) for CPs. The results show that 58% (558 of 968) of the 1996 general public believed that their knowledge regarding CPM had improved over the past 5 years.	6/7
Larue et al., (1995), France	Cross-sectional method	To assess physicians’ estimates of the prevalence of pain among patients with cancer, their practice in prescribing analgesics, their training in CPM and the quality of care received by cancer patients in their own practice and in France	900 physicians	Telephone by professional interviewers	< 35 (21.3%) for ONCs and (25.0%) for PCPs. > 45 (36.3%) for ONCs and (27.0%) for PCPs	Female oncologists (36.3%) and female primary care physician (17.0%)	Not stated	Oncologists and primary care physicians	N/A	Although 85% of primary care physicians and 93% of medical oncologists express satisfaction with their own ability to CPM, 76% of primary care physicians and 50% of medical oncologists report being reluctant to prescribe morphine for CPM. Both groups cited fear of side effects as their main reason to hesitate to prescribe morphine. Concerns about the risk of tolerance (odds ratio [OR], 1.15–2.52), perceptions that other effective drugs are available (OR, 1.11–2.41), perceptions that morphine has a poor image in public opinion (OR, 0.96–2.07), and the constraints of prescription forms (OR, 1.12–2.26) contribute significantly to physicians’ infrequent prescription of morphine, as are being female (OR, 1.01–2.03) and being an older oncologist (OR, 1.09–2.51).	4/7
Lin et al., (2000), Taiwan	Cross-sectional method	To examine attitudes held by Taiwanese family caregivers of hospice in-patients with cancer that serve as barriers to CPM; to determine the relationship of attitudinal barriers to family caregiver hesitancy to report pain and to administer analgesics; and to determine the relationship of attitudinal barriers to the adequacy of opioid used by the patient	160 palliative care patients and family caregivers	Inpatient palliative care units of two medical centres in Taipei area of Taiwan	Patients (59.63 ± 13.76); family caregivers (43.21± 12.88)	Patients, male (47%); female (53%) and family caregivers, male (27%); female (73%)	The Barriers Questionnaire–Taiwan (BQT) form, a demographic questionnaire, and the Brief Pain Inventory (BPI) Chinese version	Palliative care patients (*n* = 80) and caregivers (*n* = 80)	Lung (23%), colorectal (16%), breast (13%), liver (9%), gastric (7%), oral (6%), cervical (6%), and various other types (20%)	The five mean ± SD of BQT subscale score ranged from 0 to 5 among hospice family caregivers with the highest scores were disease progression (3.82), side-effects (3.29), p.r.n. (3.01), tolerance (2.96), and addiction (2.67), indicating that these concerns are moderately to strongly held by caregivers. Two attitudinal barriers, ‘Constipation from pain medicine is really upsetting’ and ‘Pain medicine will cause harm to kidneys’ were endorsed by 100% of caregivers. 12 of the 80 caregivers (15%) reported their hesitation to report pain in the past month. Those caregivers who had expressed hesitancy to report pain recorded significantly higher scores on the fear of addiction barrier than those who had no hesitancy. 24 of the caregivers (30%) reported that they had hesitated to administer analgesics to their patients in the past month. Those caregivers who expressed hesitancy in administering analgesics recorded significantly higher scores on the barrier items including fear of addiction, side-effects and tolerance, as well as the total BQT score, than those who had no hesitancy in administering analgesics in the past month. Older and less-educated caregivers scored significantly higher on the BOT than did their younger, more educated counterparts. 83% of these patients were classified as using adequate medication and 17% as being under-medicated.	7/7
Levin et al., (1985), Wisconsin, USA	Cross-sectional method, mixed method	To provide objective information about the public’s attitudes towards PM and the possible effects of such beliefs on a variety of factors, including delay in seeking treatment and avoidance of analgesic medications	496 general public	The Wisconsin Survey Research Laboratory	Not stated	Female (57%), male (43%)	Not stated	Adult lay public	Not stated	The result from the 472 respondents who had not been diagnosed with cancer: 15% of them agreed or strongly agreed that if they had cancer their fear of the disease would make them seeking medical care. 9% of the sample agreed or strongly agreed their concern about CP would lead to avoidance of medical care, whereas 18% indicated they would avoid seeking care as of concerns about pain associated with cancer treatment. 62% associated the onset of pain with disease progression, and 57% thought CPs usually die a painful death. 50% of respondents had significant concerns about a variety of consequences of taking opioids include confusing or disoriented, tolerance and addiction.	4/7
McCaffery and Ferrell, (1995), Australia, Canada, Japan, Spain, and the USA	Cross-sectional method	To address nurses’ knowledge and attitudes about patients’ reports of pain, prevalence of cancer pain, preferred route of administration for analgesics, preferred choice of opioid analgesic, initiation of treatment, dosing schedule, and knowledge related to addiction and use of placebos.	1428 international nurses from 5 countries	Pain programmes in Western, Eastern, Midwestern, & southern, sts in the USA, Pain programmes in Australia, pain programmes in Canada, palliative care in Japan, and from nurses had lectures In Spain	Not stated	Not stated	Not stated	Nurses in 5 countries	N/A	Prevalence of pain: higher % from nurses in Span 94.8% and lower % was only 49% of nurses in Japan. Over-reporting of pain: Nurses from Japan reported an extremely high degree of misconception, with 28.9% responding that 80–100% of CPs over report their pain. Incidence of addiction: Roughly 20–30% of nurses from each country reported the likelihood of addiction as 5%. The % was even higher of 50.9% Japanese and Spanish nurses 54.7%. Initiation of opioids: Canadian nurses reported the highest correct response with 93.2%, while was only 51.2% in Japanese nurses. Appropriate use of analgesics: widespread misconceptions in this area, with only 51.2% of nurses from Spain and 61.6% of Japan compared to 71.5% of Canadian and 66.3% American nurses who selected morphine for CPM. Reason of pt. request ↑↑ dose of opioids: Pt. was exp. ↑↑ pain, were 94.7% in Canada, whereas, only 57.8% was of Spanish nurses. Determination of pain intensity: Pt. is best judge of pain, 95.8% of Canadian nurses, while only 71.6% of Japanese nurses.	3/7
O’Brien et al., (1996), North Carolina, USA	Cross-sectional method		340 registered nurses	The North Carolina, hospital settings	52 years (range 21–73 years).	Male 3%, female 97%.	The North Carolina Cancer Pain Initiative (NCCPI) survey was adapted from the Wisconsin CPI	Registered Nurses	N/A	*Knowledge scores for the three subscale revealed that nurses who had worked with CPs were more knowledgeable than those who did not work with CPs. The total knowledge score for nurses caring for CPs was 18.47 and 15.88 for nurses not caring for CPs (t = − 6.19, p < 0.001). Attitude towards PM was for nurses caring for CPs the average was 3.52. A liberal attitude was reported more often by nurses caring for one or more CPs (X*^*2*^ *= 3.9, df = 1, p < 0.02).*	7/7
Riddell and Fitch, (1997), Canada	Descriptive correlational study	To examine patients’ knowledge of and attitudes towards cancer pain management and to identify, from patients’ perspectives, factors contributing to effective and ineffective pain relief	42 patients	Oncology facility at teaching hospital	58.5 years	Female 28 (67%), male 14 (33%)	A modified version of the Patient Pain Questionnaire (PPQ)	Cancer patients	Head/neck, breast, haematologic, female reproductive system, lung, gastrointestinal, male reproductive	The results in this study showed that many patients lacked knowledge of the principals involved in effective CPM and had unrealistic concerns about taking pain medications. Significant negative relationships were found between pain intensity rating and factors such as patients’ knowledge of PM, their level of satisfaction with pain relief and their perception of the goal of PM. Patients identified a number of impediments to effective pain relief, including concerns about addiction and various side effects to pain medications.	5/7
Shahriary et al., (2015), Iran	Cross-sectional method	To determine the baseline level of knowledge and attitudes of oncology nurses regarding CPM	58 cancer nurses	Shahid Sadoughi hospital, oncology units, Yazd, Iran	33.5 (range 25–48) years	100% female	Nurses Knowledge and Attitudes Survey Regarding Pain (NKAS) tool	Oncology nurses	N/A	The average correct response rate for oncology nurses was 66.6%, ranging from 12.1 to 94.8%. The nurses mean score on the knowledge and attitudes survey regarding PM was 28.5%. Results revealed that the mean percentage score overall was 65.7%. Only 8.6% of nurse participants obtained a passing score of 75% or greater. Widespread knowledge deficits and poor attitudes were noted in this study, particularly regard pharmacological PM.	5/7
Shahnazi et al., (2012), Iran	Cross- sectional method	To obtain information about the knowledge and attitudes of nurses concerning CPM with the use health belief model (HBM) as framework	98 nurses	Alzahra educational hospital in Isfahan, Iran	38.7 ± 7.04 years	Male 18 (18.4), female 80 (81.6)	Self-admin questionnaire designed on the basis of health belief model (HBM)	Nurses	N/A	From the 10 CP knowledge and attitude questions assessed, the mean number of correctly answered question were 61.2 (SD = 16.5) and 63 (SD = 11) with a range of 30–100 and 35–95, respectively. There was a direct correlation between knowledge and attitude of nurses with health belief model (HBM) constructs except for perceived barriers and perceived threat. Among the HBM constructs, the highest score was related to self-efficacy with mean score of 87.2 (SD = 16.4).	6/7
Srisawang et al., (2013), Thailand	Cross-sectional method	To assess the knowledge and attitudes physicians and policy makers/regulators have regarding use of opioids for CPM. Barriers to opioid availability were also studied	266 physicians and policy makers/regulators	300 hospitals in Thailand	From 36 to 45 physicians (29.2%), policy makers (27.7).	Physicians, male 126 (57.5%), female 93 (42.5%); policy makers, male 19 (40.4%), female 28 (59.5).	Not stated	Physicians (*n* = 219) and policy makers/regulators (*n* = 47).	N/A	Of the physicians, 62.1% had inadequate knowledge and 33.8% had negative attitudes. Physicians who did not know the WHO three-step ladder were more likely to have less knowledge than those having used the WHO three-step ladder (OR = 13.0, *p* < 0.001). Policy makers/regulators also had inadequate knowledge (74.5%) and negative attitudes (66.0%). Policy makers/ regulators who never had CPM training were likely to have more negative attitudes than those having had training within less than one year (OR = 35.0, *p* = 0.005). Lack of training opportunities and periodic shortages of opioids were the greatest barriers to opioid availability for physicians and policy makers/ regulators, respectively.	6/7
Utne et al., (2018), Norway	Cross-sectional method	To survey knowledge and attitudes to pain and PM among cancer care nurses, and to explore any association between various demographic variables and knowledge level	312 cancer nurses	Forum for Cancer Nursing	45 years	Female (98.4), male (1.6)	Nurses’ Knowledge and Attitudes Survey Regarding Pain (NKAS)	Norwegian oncology nurses	N/A	Norwegian nurses had a mean NKAS total score was 31 points (75%), indicating a relatively high level of knowledge and good attitudes towards pain in cancer care. Significant associations were found between NKAS total score and PM course (*p* = 0.01) and workplace (*p* = 0.04). Nurses in cancer care in Norway have relatively good pain knowledge. The potential for improvement is the greatest with regard to pharmacology and nurses’ attitudes to how patients express pain.	7/7
Vallerand et al., (2007), Detroit, Michigan, the USA	Descriptive cross-sectional method	To determine pain management knowledge and examine concerns about reporting pain and using analgesics in a sample of primary family caregivers of CPs receiving homecare	46 primary caregivers	Homecare patients with cancer	55 years (SD, 14.62 years).	Female 67.4%	The Barriers Questionnaire, the Family Pain Questionnaire	Primary caregivers	N/A	The mean for each subscale of the BQ of caregivers expressing some agreement of concerns between 1.05 and 2.41. The concerns were barriers to reporting pain and using analgesics, and up to 15% reported having strong agreement. The areas of greatest concern were about opioid related side effects (2.41), fears of addiction (2.35), the belief that pain meant disease progression (2.28), and tolerance (1.37). Results showed that caregivers with higher PM knowledge had significantly fewer barriers to CPM, supporting the importance of increasing caregiver’s knowledge of CPM.	7/7
Von Roenn et al., (1993), USA	Cross-sectional method	To determine the amount of knowledge about CPM among physicians practicing in ECOG-affiliated institutions and to determine the methods of pain control being used by physicians	897 physicians	The Eastern Cooperative Oncology Group (ECOG).	Not stated	Not stated	Physician cancer pain questionnaire	Physicians with patient care (oncologists, haematologists, surgeons and radiation therapists)	N/A	Concerning the use of analgesics for cancer pain in the United States (*n* = 864), 86% of the respondents thought that the majority of patients with pain are under-medicated, although 13% thought that most patients receive adequate treatment for pain. Most of the sample (67%) thought that at least 50% of the cancer patients they treat had pain at some point during their illness. Physicians estimated that almost one half of cancer patients (48%) had pain for more than 1 month.	7/7
Wells et al., (2001), Scotland, UK	Cross-sectional method	To assess the knowledge and attitudes of nursing and medical staff working in a surgical unit, before and after working with a newly established Hospital PC team	101 nursing and medical staff	A surgical unit, hospital palliative care team	34 years	Male 22 (22%) and female 79 (78%)	Not stated	Physicians (*n* = 22) and nurses (*n* = 79)	N/A	At baseline, 24% of staff showed a lack of knowledge and a negative attitude towards the risk of addiction to morphine. Regarding opioid tolerance, at the follow-up time point, only 14% demonstrating a lack of knowledge. At follow-up, 34% (compared with 50% at baseline) still believed that increased doses of opioids were needed because opioids became ineffective over time. Although 25% of all staff still lacked knowledge about the risk of respiratory depression at follow-up, this was a significant improvement on the 56% who demonstrated a lack of knowledge at baseline. At baseline, a fairly high proportion of staff appeared to believe pain was always a part of advanced cancer (38%).	4/7
Yanjun et al., (2010), China	Survey	To determine the degree of physician knowledge on morphine use and the factors that impede morphine use in clinical practice in China.	201 physicians	4 hospitals in China	Not stated	Not stated	Not stated	Physicians	N/A	Physicians who reported having received training in CPM and drug use demonstrated a significantly higher mean score of basic knowledge compared to physicians who reported not having received training (9.31 ± 2.88:8.23 ± 2.70, *u* = 2.74,*p* < 0.001). The top three cited impediments to widespread clinical use of morphine for cancer pain were: (1) lack of professional knowledge and training (57.2%); (2) fear of opioid addiction (48.7%); and (3) physicians’ personal preferences to select other drugs (46.0%).	6/7
Yildirim et al., (2008), Turkey	Cross-sectional method	To examine information about the knowledge and attitudes of Turkish oncology nurses regarding CPM	68 oncology nurses	Oncology& haematology units in two university hospitals located in Izmir, Turkey	From 21 to 30 years	Not stated	Knowledge and Attitudes Survey Regarding Pain (NKASRP)	Oncology nurses	N/A	The findings showed that Turkish oncology nurses have insufficient knowledge and attitudes about CPM which is widely recommended by the WHO. Out of the 39 pain questions examined, the mean score for correctly answered items was 13.81 (35.41% correct answer rate). Compared with earlier research using the same tool. Only 8.8% of oncology nurses correctly identify that less than 1% of patients who receive opioids far pain relief will develop addiction, and 91.2% erroneously believe that addiction will occur in patients. Most nurses (97.1%) incorrectly believed that more patients over-report their pain.	7/7
Zhang et al., (2015), China	Cross-sectional method	To evaluate physicians’ current practice, attitudes towards, and knowledge of cancer pain management in China	500 physicians	11 medical facilities in China	< 35–≥ 35 years	Male (*n* = 212, 45.4%), female (*n* = 255, 54.6%).	Not stated	Physicians (oncologists, internists, haematologists)	N/A	About 32.6% of physicians assessed patients’ pain rarely, and 85.5% never or occasionally treated patients’ cancer pain together with psychologists. More than 50% of physicians indicated that opioid dose titration in patients with poor pain control and assessment of the cause and severity of pain were urgently needed knowledge for CPM. Inadequate assessment of pain and PM (63.0%), patients’ reluctance to take opioids (62.2%), and inadequate staff knowledge of PM (61.4%) were the three most frequently cited barriers to physicians’ CPM.	4/7

### Overall Results of Included Studies

#### Patients’ Knowledge and Attitudes Towards CPM

The results from the majority of studies with cancer patients reported that the mean scores on patient’s knowledge and attitudes towards CPM were low, indicating poor understanding or negative attitudes towards CPM [19, 20, 57, 77]. For example, a recent study conducted in China by Lou and Shang [57] reported through the Barriers Questionnaire-Taiwan (BQT; ranged from 0 to 5) that patients had negative attitudes towards CPM in six areas (scores ≥ 2.5), “tolerance” (3.83 ± 0.96), “use of analgesics as needed (p.r.n.)” (3.73 ± 1.01), “addiction” (3.44 ± 1.05), “disease progression” (3.28 ± 1.26), “distraction of physicians” (3.16 ± 1.07) and “side effects” (2.99 ± 0.68), which can lead to attitudinal barriers towards effective CPM [2]. Another example [20] is that more than 50% of Turkish patients refused to receive strong opioids, such as morphine, and 36.8% of them preferred another (non-opioid) medication for managing their cancer pain.

### Professionals’ Knowledge and Attitudes Towards CPM

Several studies showed that physicians had better knowledge and attitudes towards CPM compared with nurses [22, 32, 44, 45]. For instance, it has been reported that physicians who work at oncology units had higher understanding and knowledge about CPM than nurses. The mean scores on the KAS (range 0–39) for physicians was 24.3 (62.3%) compared with 20.08 (51.5%) for nurses (*p* < 0.001) [22]. The outcomes also showed that oncologists recorded higher knowledge of CPM than surgeons (*p* < 0.001) [33]. An interesting finding, which was reported by McCaffery and Ferrell [59], is that Canadian and American nurses were more likely to use morphine for CPM than nurses in Japan or Spain. For example, 71.5% of Canadian nurses and 66.3% of nurses from America reported using morphine for managing cancer pain, compared with 61.6% of Japanese nurses and 51.2% of nurses from Spain [59]. The results also revealed that there was a degree of misunderstanding regarding opioid addiction by nurses between countries. For instance, the majority of nurses who answered the relevant questions correctly were from Canada and the USA (51.3% and 43.4%), respectively, whereas, only 14% of Spanish nurses and 17.2% of Japanese nurses responded correctly [59]. Another interesting observation to emerge from the results was that there were geographical variations within countries, for example, the nurses who worked in the central region of Italy had lowest score of pain knowledge (47.9%; M = 18; *n* = 66) compared with those in the north (57.2%; *M* = 21; *n* = 149) and in the south of Italy (56.9%; *M* = 23; *n* = 72) (*p* < 0.001) [11].

### Family Caregivers’ Knowledge and Attitudes Towards CPM

A study revealed that caregivers’ attitudes towards CPM and the patients’ pain knowledge explained 23.2% of the total variance in the patients’ average scores for their attitudes towards CPM when entered into a regression equation [57]. This indicates that patients’ attitudes towards CPM were influenced by their caregivers’ attitudes and the patient’s pain knowledge [57]. The results from a study conducted in Taiwan indicated that family caregivers held some moderate to strong concerns towards CPM. These concerns were shown through the Barriers Questionnaire-Taiwan (BQT) survey (ranged 0–5) as follows: disease progression (3.82), side effects (3.29), given as needed (p.r.n) (3.01), tolerance (2.96) and addiction (2.67) [55]. The results also showed some family caregivers reporting their hesitation to administer opioids and to report pain to their patients during the preceding month, because caregivers believed that opioids would cause constipation and harm to patients’ kidneys [55]. Surprisingly, there were also similar concerns towards CPM by caregivers in China, where these concerns were shown as higher or lower in some dimensions; tolerance (3.74), given as needed (p.r.n) (3.51), addiction (3.43), disease progression (3.27) and side effects (3.22) [57]. However, these concerns were lower in the USA, indicating that caregivers in the USA might have a good level of knowledge and positive attitudes towards CPM compared with caregivers in Taiwan and China. For example, the areas of concern for caregivers in the USA were about opioid-related side effects (2.41), fears of addiction (2.35), disease progression (2.28) and tolerance (1.37) [95].

### General Public’s Knowledge and Attitudes Towards CPM

The results from 472 general public respondents in the USA who had not been diagnosed with cancer showed that 18% indicated they would avoid seeking care because of concerns about pain associated with cancer treatment. Fifteen percent of the sample agreed or strongly agreed if they had cancer their fear of the disease would make them seek medical care, whereas 9% of them agreed or strongly agreed their concern about cancer pain would lead to avoidance of medical care [51]. The most common key concern among the general public in the USA that would affect them if they had cancer was the “potential for upset to their family”, followed by concern about the “possibility of dying of cancer”. Nearly 50% reported a significant concern about pain resulting from both the cancer and the process of its management [51]. The study also reported that 62% of the general public believed that pain is usually associated with disease progression, 57% thought that cancer patients usually die with a painful death and 50% had significant concerns about opioid side effects including confusion or disorientation, tolerance and opioid addiction [51].

## Discussion

We aimed to systematically review research on the nature and impact of attitudes and knowledge towards CPM. Overall, the results of this review show that a majority of included studies indicated similar attitudinal barriers to effective CPM shared across patients, caregivers, professionals and the public. The barriers most commonly cited by professionals [11, 22, 28, 44, 48, 59, 98, 100], patients and their caregivers [20, 55, 57, 95] and the general public [51] were the fear of poor tolerance, side effects of opioids and drug addiction. However, the most common barriers cited by professionals were contrary to other similar studies, which have suggested that the most important barriers were poor assessment of pain and its management, patient reluctance to take opioids and inadequate staff knowledge of CPM [14, 22, 27, 32, 34, 44, 45, 104]. Furthermore, a previous systematic review by Jacobsen et al. [42] showed that physicians from countries, such as some states in the USA, Australia and Denmark were more often prescribing strong opioids in efficient doses, as they were less concerned about opioid addiction [42]. Nonetheless, their general findings were that physicians consistently reported being concerned about high doses of opioid and the fear of side effects, and these fears were common reasons for reluctance to prescribe adequate amounts of opioids for managing cancer pain [42]. It can thus be suggested that people from different countries have different attitudes and knowledge towards CPM.

One interesting finding was that the results from the majority of studies with cancer patients showed low mean scores on patient’s knowledge and attitudes towards CPM [19, 20, 57, 77]. This result may be explained by the fact that many patients could be reluctant to report their pain to professionals because they have a mistaken belief regarding opioid medication [68]. This finding was also reported by a systematic exploratory review by Jacobsen et al. [43]. Another important finding was that negative attitudes towards morphine were shown by Turkish patients as they continued rejecting morphine for their cancer pain after sessions about opioids were given. The reasons for that were due to fear of addiction, religious reasons and cultural prohibitions [20, 58]. Silbermann and Hassan [88] stated that patients’ response to cancer can differ based on the patients’ beliefs and culture. It has been argued that many patients and their family caregivers viewed opioid medications as a path to death; accordingly, opioid analgesics became their last choice [87]. Despite pain being considered an individual experience, many patients are influenced by their culture, mainly when they are interpreting their pain or accepting the medication of CPM [5, 23, 65]. Therefore, understanding patients’ culture and beliefs can provide the professional with a consideration into how cancer is viewed by the patient [88]. However, professionals can also be influenced by their culture, as it has been reported that cultural beliefs among professionals were one of the most obviously identified barriers towards CPM [80].

Another interesting outcome was that several studies showed physicians had a better level of attitudes and knowledge towards CPM than nurses [22, 32, 44, 45]. There was also a difference between oncologists and surgeons regarding their level of knowledge about cancer pain and its management [33]. It seems possible that these results are due to work experience and training in CPM, as many studies have shown that working with cancer patients’ care and receiving training in CPM can improve professionals’ knowledge and attitudes towards CPM [29, 38, 40, 44, 50, 67, 94, 100].

Most notably, there was a variation between nurses from different countries regarding the level of knowledge and attitudes towards CPM [59]. As could be expected, the variation in knowledge about CPM among those nurses could indicate that morphine is under-prescribed. This view was supported by a systematic review by Oldenmenger et al. [68] who reported that the rates of adherence to opioids for CPM varied from 20 to 95%, with the majority of cancer patients taking their treatments only as needed.

The results also showed that some oncology nurses had an incorrect self-evaluation about their knowledge in CPM [11, 102]. This finding is consistent with that of Omran et al. [70] who also found that Jordanian oncology and non-oncology nurses have a low level of knowledge about CPM. In contrast to earlier findings, several studies indicated that the oncology nurses and doctors achieved higher scores on the knowledge and attitudes surveys (KAS) compared with general nurses and physicians [33, 44, 50, 84, 94]. These positive results could be due to the work experience of professionals in cancer pain settings, as this was reported by McCaffery and Ferrell [59] who stated that nursing staff from countries such as Canada and the USA, which have the longest experience of palliative care units, showed a better level of attitudes and knowledge about CPM than nurses from countries (Japan and Spain) that had palliative care services more recently.

However, it seems that direct experience in oncology units without education and training is not enough to increase professionals’ knowledge about CPM. This view was supported by Bernardi et al. [11] who reported that the years of experience of cancer nurses were not related to pain knowledge scores (*p* = 0.2). It is possible therefore that education in CPM is the key issue for improving the professionals’ level of knowledge and attitudes towards CPM. A number of authors have considered the effects of educational interventions on professionals’ attitudes and knowledge towards CPM [4, 6, 9, 12, 49, 70, 71]. According to previous systematic reviews of educational interventions aimed to improve CPM in different settings, a significant effect was shown on pain scores, however, the quality of opioid prescription and interference from pain in daily activities was not affected by the majority of interventions [4, 6, 8, 69].

As could be expected, lack of professional education and training in CPM could be one of the most important key barriers for physicians and nurses [34, 39]. Furthermore, this was reported as the highest physician barrier to morphine usage in clinical practice [100]. Another argument was that professionals with cancer patients’ care need professional teaching regarding CPM, which could aid patients in reporting pain and in effectively using the opioids that are prescribed to them [39, 97]. It is also well documented that there is less than optimal pain management for patients with cancer as a result of a lack of professional healthcare education about CPM [18, 60]. Numerous studies have showed that professionals who had experience in palliative care units, receiving training and high level of education in CPM obtained higher scores on the knowledge of cancer pain and its management [45, 49, 70, 71, 94, 100].

Several studies have shown that caregivers had low level of knowledge and attitudes towards CPM [55, 57, 95]. These negative attitudes and inadequate knowledge by caregivers towards opioids could result in attitudinal barriers towards effective CPM [30, 54, 55]. Therefore, it has been argued that it is important to increase caregivers’ ability to participate in CPM and enable them to assess pain and to help their patients take adequate doses of opioids [101]. The correlation between caregivers’ attitudes and their patients’ pain knowledge towards CPM is interesting because patients’ attitudes towards CPM were influenced by their caregivers’ attitudes and the patient’s pain knowledge [57]. Therefore, caregivers should have general awareness and adequate level of knowledge about CPM. It has been argued that caregivers with higher pain management knowledge had significantly fewer barriers to CPM [95].

Results from a study on the general public showed that many people were concern about disease progression and believed that pain was usually associated with this concern. However, some of the public had significant concerns about opioids side effects, tolerance and addiction [51]. Surprisingly, only two studies were found on the general public’s attitudes and knowledge towards CPM and both of articles were published before 2000, consequently updated studies about this area are needed.

Overall, the results of this review have found some evidence that there are negative attitudes and lack of knowledge towards CPM among the four groups included in this review. These findings are consistent with those of recent studies and systematic reviews [12, 13, 20, 26, 37, 79, 80, 96]. Thus, it can be argued that due to these negative attitudes and lack of knowledge towards CPM, the management of cancer pain remains a major problem worldwide, especially in countries within Europe, Africa and Asia [13, 26, 52, 75, 76, 81, 92]. These could be due to lack of education and training about CPM among professionals and lack of general awareness and adequate level of knowledge about CPM among patients, caregivers and the public, as these were stated in all of the included studies. Therefore, healthcare professionals expressed a desire for additional education and training on CPM. A recent systematic review indicated that educational programmes on CPM, including CPM topics in nursing curricula, and training programmes on CPM are the most important factors for enhancing nurses’ knowledge and attitudes towards CPM [12]. It has also been argued that nurses who had received educational programmes on CPM reported significantly higher mean of scores on knowledge about CPM than those who did not have pain education (*M* = 22 versus *M* = 20; *p* = 0.02) [11].

Furthermore, patients, caregivers and the public need general awareness and adequate level of knowledge about CPM. A systematic review reported that providing educational sessions on CPM can improve caregivers’ knowledge and reduce their attitudinal barriers towards CPM [62]. Regarding the general public’s views, it is expected and inevitable that the general public will know very little about CPM unless they have cancer or someone close to them does. Thus, general awareness and adequate level of knowledge about CPM are needed.

### Limitations

As only studies published in English were considered within the inclusion criteria, as well as just published studies, it is possible that there are studies that have been published in other languages, also unpublished articles that could have been included in this review. Other limitations could be that even though all included studies used the same design (cross-sectional design), the questionnaires that were used to conduct surveys in this particular area were different and some studies did not state which questionnaire was used or failed to provide information regarding the validity of the tools. Therefore, it was difficult to directly compare studies and the reliability of these included studies in this review could be compromised [74, 90]. In the quality analysis, 15 of the 36 included studies were judged to be only fair quality (see Appendix 3). The reason for a fair quality score instead of a good quality score is that these articles had some methodological limitations. However, almost two-thirds of the included studies, 25 out of the 36 (69.44%), were rated as of good quality. Included studies were from high and low income countries and thus different healthcare systems and cultural beliefs across people form these countries could have affected their attitudes and knowledge towards CPM. Moreover, the possibility of bias could have happened during the reporting of outcomes.

### Implications for Clinical Practice

Healthcare professionals should follow specific guidelines for CPM, which have been established by WHO [91] and NICE [10, 66]. Moreover, knowledge and attitudes of professionals need to be improved by intensive training on opioids and educational interventions about cancer pain and its management in order to have effective CPM. Likewise, patients, caregivers and the public will need different approaches to improve general awareness and obtain an adequate level of knowledge about CPM.

### Implications for Research

All studies included in this review were quantitative studies. More in-depth understanding of the conceptions and attitudes towards CPM can be provided by qualitative studies [93]. Additionally, qualitative methods could help to identify the factors which can influence the professionals, cancer patients, caregivers and the general public’s attitudes and knowledge towards CPM [93]. Furthermore, more updated studies within CPM are needed to generate more contemporary data in this area.

## Conclusions

This systematic review confirms that there are still barriers to effective CPM by professionals, patients, caregivers and the general publics’ lack of knowledge and/or poor attitudes towards CPM, which might result in unalleviated cancer pain. More detailed understanding of how these attitudes arise within different contexts and tailoring educational initiatives to address these are likely to have most impact on improving CPM.

## Electronic Supplementary Material

ESM 1 (DOCX 137 kb)
