# Supplementary material for: Managing Pain in People with Cancer—a Systematic Review of the Attitudes and Knowledge of Professionals, Patients, Caregivers and Public
Source: J Cancer Educ. 2019 May 22;35(2):214–40. doi: 10.1007/s13187-019-01548-9 (PMC7076060; doi:10.1007/s13187-019-01548-9)
Supplement: Supplementary file 1 — (DOCX 137 kb) [file 13187_2019_1548_MOESM1_ESM.docx]

Supplementary materials

**Managing Pain in People with Cancer– a Systematic Review of the attitudes and knowledge of professionals, patients, caregivers and public**

##### Appendix 1 Data extraction form

| **Data extraction form** | | |
| --- | --- | --- |
| **General information** | **Extracted data** | **Comments** |
| First author |  |  |
| Article title |  |  |
| Year of study |  |  |
| Country |  |  |
| Type of publication |  |  |
| **Study characteristics** |  |  |
| Study design | Cross-sectional Cohort ☐Case-Control  ☐Qualitative mixed methods |  |
| Study aim |  |  |
| Study setting |  |  |
| Inclusion criteria |  |  |
| Exclusion criteria |  |  |
| Type of sample | Professionals or cancer patients or caregivers or the general public. |  |
| Measurements |  |  |
| **Participant characteristics** |  |  |
| Number of participants |  |  |
| Type of conditions |  |  |
| Mean age |  |  |
| Range of age |  |  |
| Gender |  |  |
| **Study outcomes** | Attitudes and knowledge towards cancer pain management |  |
| Attitudes | Professionals, patients with cancer, caregivers, general public |  |
| Knowledge | Professionals patients with cancer, caregivers, general public |  |
|  |  |  |
| **Other relevant findings** |  |  |
|  |  |  |
|  |  |  |
|  |  |  |

##### Appendix 2 The Joanna Briggs Institute Analytical Cross Sectional Studies Assessment (JBI-ACSSA) tool.

**JBI Critical Appraisal Checklist for Analytical Cross Sectional Studies**

Reviewer……………………… Date………………………………………………………….

Author ……………………………….Year…………….. Record Number………………….

Yes No Unclear Not

Applicable

1. Were the criteria for inclusion in the sample clearly defined? □ □ □ □

2. Were the study subjects and the setting described in detail? □ □ □ □

3. Was the exposure measured in a valid and reliable way? □ □ □ □

4. Were objective, standard criteria used for measurement of □ □ □ □

the condition?

5. Were confounding factors identified? □ □ □ □

6. Were strategies to deal with confounding factors stated? □ □ □ □

7. Were the outcomes measured in a valid and reliable way? □ □ □ □

8. Was appropriate statistical analysis used? □ □ □ □

Overall appraisal: Include □ Exclude □ Seek further info □

Comments (Including reason for exclusion)

---------------------------------------------------------------------------------------------------------------------------------------------------------------------------------------------------------------------------------------------------------------------------------------

© Joanna Briggs Institute 2017

##### Appendix 3: Appraisal of methodological quality of 36 included studies

|  | Methodological items based on JBI-ACSSA tool | | | | | | | | Score |
| --- | --- | --- | --- | --- | --- | --- | --- | --- | --- |
|  | 1 | 2 | 3 | 4 | 5 | 6 | 7 | 8 |  |
| Author (s), year, and Country | Inclusion  Criteria defined? | Subjects and setting described in detail? | Exposure measured reliable/valid? | Measurement of condition  Standard? | Confounding factors identified? | Confounding factors addressed? | Outcomes  Measured in a valid way? | Statistical analyses appropriate? |  |
| Bernardi et al., (2007). Italy. | + | + | N/A | + | + | + | - | + | 6/7 |
| Breuer et al., (2011). New York, the US. | + | + | N/A | + | - | - | - | + | 6/7 |
| Cohen et al., (2005). Israel. | + | + | N/A | + | + | + | + | + | 7/7 |
| Colak et al., (2014). Turkey. | + | + | N/A | + | + | + | - | + | 6/7 |
| Darawad et al., (2017). Jordan. | + | + | N/A | + | - | - | + | + | 5/7 |
| Eftekhar et al., (2007). Iran. | + | + | N/A | + | - | - | - | + | 4/7 |
| Elliott et al, (1996). The US. | + | + | N/A | + | - | - | - | + | 6/7 |
| Elliott et al., (1992). State of Minnesota, the US. | + | + | N/A | + | + | + | + | + | 7/7 |
| Elliott et al., (1995). The US. | + | + | N/A | + | - | - | + | + | 7/7 |
| Furstenberg et al., (1998). State of new Hampshire, the US. | + | + | N/A | + | + | + | - | + | 6/7 |
| Gallagher et al., (2004).British Columbia. | + | + | N/A | + | + | + | - | + | 6/7 |
| Ger et al., (2000). Taiwan | + | + | N/A | + | + | + | - | + | 6/7 |
| Hollen et al., (2000). South Central State. The US. | + | + | N/A | + | - | - | + | + | 5/7 |
| Jeon et al., (2007). Korea. | + | + | N/A | + | + | + | - | + | 6/7 |
| Jho et al., (2014). Korea. | + | + | N/A | + | + | + | - | + | 6/7 |
| Kaki, (2011). Saudi Arabia. | + | + | N/A | + | - | - | - | + | 4/7 |
| Kassa and Kassa, (2014). Ethiopia. | + | + | N/A | + | + | - | + | + | 6/7 |
| Kim et al., (2011). South Korea. | + | + | N/A | + | + | + | + | + | 7/7 |
| Kuzeyli Yildirim et al., (2008). Turkey. | + | + | N/A | + | - | - | + | + | 7/7 |
| Larue et al., (1995). France. | + | + | N/A | + | - | - | - | + | 4/7 |
| Larue et al., (1999). France. | + | + | N/A | + | + | + | - | + | 6/7 |
| Levin et al., (1985). Wisconsin, the US. | + | + | N/A | + | - | - | - | + | 4/7 |
| Lin et al., (2000). Taiwan. | + | + | N/A | + | + | + | + | + | 7/7 |
| Lou and Shang, (2017). China. | + | + | N/A | + | + | + | + | + | 7/7 |
| McCaffery and Ferrell, (1995). Australia, Canada, Japan, Spain, and the US. | + | + | N/A | + | - | - | - | - | 3/7 |
| O’Brien et al., (1996). North Carolina, the US. | + | + | N/A | + | - | - | + | + | 7/7 |
| Riddell and Fitch, (1997). Canada. | + | + | N/A | + | - | - | + | + | 5/7 |
| Shahnazi et al., (2012). Iran. | + | + | N/A | + | + | + | - | + | 6/7 |
| Shahriary et al., (2015). Iran. | + | + | N/A | + | - | - | + | + | 5/7 |
| Srisawang et al., (2013). Thailand. | + | + | N/A | + | + | + | - | + | 6/7 |
| Utne et al., (2018). Norway. | + | + | N/A | + | + | + | + | + | 7/7 |
| Vallerand et al., (2007). Detroit, | + | + | N/A | + | + | + | + | + | 7/7 |
| Von Roenn et al., (1993). The US. | + | + | N/A | + | + | + | + | + | 7/7 |
| Wells et al., (2001). Scotland, the UK. | + | + | N/A | + | - | - | - | + | 4/7 |
| Yanjun et al., (2010). China. | + | + | N/A | + | + | + | - | + | 6/7 |
| Zhang et al., (2015). China. | + | + | N/A | + | - | - | - | + | 4/7 |

Note: + indicates satisfies criteria, – indicated does not satisfy criteria, N/A = Data Not Available.

**Source:** Adapted from Poudel et al. [72].

##### Appendix 4 List of excluded studies with reasons

**Not related to attitudes or knowledge: n= 42**

ALSIRAFY, S. A., R. N. SALEH, R. FAWZY, A. A. ALNAGAR, A. M. HAMMAD, W. EL-SHERIEF, D. E. FARAG and R. H. RADWAN. 2015. The fear of using tramadol for pain control (tramadolophobia) among Egyptian patients with cancer. Journal of Opioid Management, 11(6), pp.474-80.

ARANDA, S., P. YATES, H. EDWARDS, R. NASH, H. SKERMAN and A. MCCARTHY. 2004. Barriers to effective cancer pain management: a survey of Australian family caregivers. European Journal of Cancer Care, 13(4), pp.336-343.

BERRY, P. E. and S. E. WARD. 1995. Barriers to pain management in hospice: a study of family caregivers. Hospice Journal - Physical, Psychosocial, & Pastoral Care of the Dying, 10(4), pp.19-33.

BERTRAM, L., S. STIEL, F. ELSNER, L. RADBRUCH, A. DAVIES, F. NAUCK and B. ALT-EPPING. 2010. [Experiences of cancer patients with breakthrough pain and pharmacological treatments]. Der Schmerz, 24(6), pp.605-12.

BUCHER, J. A., G. B. TROSTLE and M. MOORE. 1999. Family reports of cancer pain, pain relief, and prescription access. Cancer Practice, 7(2), pp.71-7.

CHOI, Y. S., S. H. KIM, J. S. KIM, J. LEE, J. H. KANG, S. Y. KIM, C. S. KIM, H. S. SONG, H. Y. LIM, C. Y. YIM, I. J. CHUNG, G. J. CHO, M. A. LEE, J. PARK and C. H. YEOM. 2006. - Change in Patients' Satisfaction with Pain Control After Using the Korean Cancer Pain Assessment Tool in Korea. Journal of Pain and Symptom Management, 31(6), pp.553-562.

DAYER, L., H. S and M. B. 2018. - A Palliative Care Clinic's Experience with Medication Adherence to Neuropathic Pain Medications. Journal of Palliative Medicine, 21(2), pp.245-248.

HODGE, F., K. NANDY, M. CADOGAN, T. ITTY, U. WARDA, F. MARTINEZ and A. QUAN. 2016. Predictors of pain management among American Indian cancer survivors. Journal of Health Care for the Poor and Underserved, 27(2), pp.636-643.

HOJSTED, J., O. EKHOLM, G. P. KURITA, K. JUEL and P. SJØGREN. 2013. Addictive behaviors related to opioid use for chronic pain: a population-based study. Pain [online]. 154(12), pp.2677-2683. Available from: <http://cochranelibrary-wiley.com/o/cochrane/clcentral/articles/248/CN-01122248/frame.html>.

HOVI, S. L. and S. LAURI. 1999. Patients' and nurses' assessment of cancer pain. European Journal of Cancer Care, 8(4), pp.213-9.

HOVIND, I. L., I. S. BREDAL and A. DIHLE. 2013. Women's experience of acute and chronic pain following breast cancer surgery. Journal of Clinical Nursing, 22(7-8), pp.1044-52.

FITCH, M. I., A. MCANDREW and S. BURLEIN-HALL. 2013. A Canadian online survey of oncology nurses' perspectives on the management of breakthrough pain in cancer (BTPc). Canadian Oncology Nursing Journal, 23(1), pp.28-43.

JACOBSEN, R., J. SAMSANAVICIENE, Z. LIUABARSKIENE and A. SCIUPOKAS. 2010. Barriers to pain management among Lithuanian cancer patients. Pain Practice, 10(2), pp.145-57.

JACOBSEN, R., J. SAMSANAVICIENE, Z. LIUBARSKIENE, P. SJOGREN, C. MOLDRUP, L. CHRISTRUP, A. SCIUPOKAS and O. B. HANSEN. 2014. Barriers to cancer pain management in Danish and Lithuanian patients treated in pain and palliative care units. Pain Management Nursing, 15(1), pp.51-8.

KIM, B. S. and H. C. CHUNG. 1991. Experience with a controlled-release oral morphine for cancer pain management. Postgraduate Medical Journal, 67 Suppl 2, pp.S82-6.

KINOSHITA, S., M. MIYASHITA, T. MORITA, K. SATO, T. MIYAZAKI, A. SHOJI, Y. CHIBA, S. TSUNETO and Y. SHIMA. 2016. Changes in Perceptions of Opioids before and After Admission to Palliative Care Units in Japan: Results of a Nationwide Bereaved Family Member Survey. American Journal of Hospice & Palliative Medicine, 33(5), pp.431-8.

KOLLER, A., C. MIASKOWSKI, S. GEEST, O. OPITZ and E. SPICHIGER. 2012. Patients' and family caregivers' experiences with a self-management intervention for cancer pain. Palliative medicine. [online]. 26(4), pp.456-457. Available from: <http://cochranelibrary-wiley.com/o/cochrane/clcentral/articles/295/CN-01025295/frame.html>.

KWON, J. H., H. D, C. G and B. E. 2013a. - Predictors of long-term opioid treatmentamongpatientswho receive chemoradiation for head and neck cancer. Oncologist, 18(6), pp.768-774.

KWON, J. H., H. D, C. G, H. W.T, N. L and B. E. 2013b. - Experience of barriers to pain management in patients receiving outpatient palliative care. Journal of Palliative Medicine, 16(8), pp.908-914.

LEBARON, V., BECK, S. L., MAURER, M., BLACK, F. & PALAT, G. 2014. An Ethnographic Study of Barriers to Cancer Pain Management and Opioid Availability in India. Oncologist, 19, 515-522.

LIN, C. C. & WARD, S. E. 1995. Patient-related barriers to cancer pain management in Taiwan. Cancer Nursing, 18, 16-22.

LETIZIA, M., CREECH, S., NORTON, E., SHANAHAN, M. & L., H. 2004. - Barriers to caregiver administration of pain medication in hospice care. Journal of Pain and Symptom Management, 27, 114-124.

MEEKER, M. A., D. FINNELL and A. K. OTHMAN. 2011. Family caregivers and cancer pain management: a review. Journal of Family Nursing, 17(1), pp.29-60.

MEGHANI, S. H. and G. J. KNAFL. 2017. Salient concerns in using analgesia for cancer pain among outpatients: A cluster analysis study. World Journal of Clinical Oncology, 8(1), pp.75-85.

MONTESANO, T., S. GIACOMOBONO, G. ACQUALAGNA, M. COLANDREA, A. DI NICOLA, L. TRAVASCIO, M. GIANCAMERLA, R. D'APOLLO, M. TOTEDA, F. UGOLINI, M. FILESI and G. RONGA. 2009. Our experience on pain palliation of bone metastasis with Sr-89 or Sm-153 in cancer patients resistant to a conventional analgesic therapy. A retrospective study. Clinica Terapeutica, 160(3), pp.193-9.

NERON, S., S. PEREZ, R. BENC, A. BELLMAN, Z. ROSBERGER and T. VUONG. 2014. - The experience of pain and anxiety in rectal cancer patients during high-dose-rate brachytherapy. Current Oncology, 21(1), pp.e89-e95.

OGASAWARA, C., Y. KUME and M. ANDOU. 2003. Family satisfaction with perception of and barriers to terminal care in Japan. Oncology Nursing Forum, 30, pp.E100-5.

OLIVER, D. P., W.-L. E, D. G, W. K, P. D and D. M. 2008. Barriers to Pain Management: Caregiver Perceptions and Pain Talk by Hospice Interdisciplinary Teams. Journal of Pain and Symptom Management, 36(4), pp.374-382.

OSIPOVA, N. A., G. A. NOVIKOV, M. S. VETSHEVA, B. M. PROKHOROV, V. A. BERESNEV, N. A. LOSEVA, S. ZEMSKAIA and T. A. SMOLINA. 1994. [First experience in the use of a new Russian narcotic analgesic prosidol in oncology]. Anesteziologiia i Reanimatologiia, (4), pp.53-7.

PAICE, J. A., T. C and S. S. 1998. - Barriers to cancer pain relief: Fear of tolerance and addiction. Journal of Pain and Symptom Management, 16(1), pp.1-9.

PANTELI, V. and E. PATISTEA. 2007. Assessing patients' satisfaction and intensity of pain as outcomes in the management of cancer-related pain. European Journal of Oncology Nursing, 11(5), pp.424-33.

PAYNE, R., S. D. MATHIAS, D. J. PASTA, L. A. WANKE, R. WILLIAMS, R. MAHMOUD, R. PAYNE, S. D. MATHIAS, D. J. PASTA, L. A. WANKE, R. WILLIAMS and R. MAHMOUD. 1998. Quality of life and cancer pain: satisfaction and side effects with transdermal fentanyl versus oral morphine. Journal of Clinical Oncology, 16(4), pp.1588-1593.

PERETTI-WATEL, P., M. K. BENDIANE, A. GALINIER, J. M. LAPIANA, R. FAVRE, H. PEGLIASCO, Y. OBADIA and G. SOUTH-EASTERN FRANCE PALLIATIVE CARE. 2004. Opinions toward pain management and palliative care: comparison between HIV specialists and oncologists. AIDS Care, 16(5), pp.619-27.

POTTER, V. T., C. E. WISEMAN, S. M. DUNN and F. M. BOYLE. 2003. Patient barriers to optimal cancer pain control. Psycho-Oncology, 12(2), pp.153-60.

RANKIN, M. A. and B. SNIDER. 1984. Nurses' perceptions of cancer patients' pain. Cancer Nursing, 7(2), pp.149-55.

RAUCK, R., N. PARIKH, L. DILLAHA, J. BARKER and L. STEARNS. 2015. Patient Satisfaction with Fentanyl Sublingual Spray in Opioid-Tolerant Patients with Breakthrough Cancer Pain. Pain Practice, 15(6), pp.554-63.

SILVONIEMI, M., T. VASANKARI, T. VAHLBERG, E. VUORINEN, K. E. CLEMENS and E. SALMINEN. 2012. Physicians' self-assessment of cancer pain treatment skills--more training required. Supportive Care in Cancer, 20(11), pp.2747-53.

TORRENT, J. C., C. J. SÁNCHEZ, J. S. ORTIZ, N. B. LÓPEZ, C. C. HERRERO, J. C. ESPINOSA, J. L. GINER, J. M. SALCEDO, K. F. WARNER, M. G. MONTOYA and E. D.-R. GARCÍA. 2007. Oncologists’ perceptions of cancer pain management in Spain: The real and the ideal. European Journal of Pain, 11(3), pp.352-359.

THINH, D. H. Q., W. SRIRAJ, M. MANSOR, K. H. TAN, C. IRAWAN, J. KURNIANDA, Y. P. NGUYEN, A. ONG-CORNEL, Y. HADJIAT, H. MOON and F. O. JAVIER. 2018. Patient and Physician Satisfaction with Analgesic Treatment: Findings from the Analgesic Treatment for Cancer Pain in Southeast Asia (ACE) Study. Pain Research and Management, 2018, p8.

**Not related to cancer pain or opioid: n= 35**

AL-EASSA, A. A., A. M. AL-FADEL, M. A. AL-AJMI, A. A. AL-NAJJAR, G. M. MAKBOUL and M. ELSHAZLY. 2012. - Knowledge and attitude of primary care doctors towards management of postmenopausal symptoms. Alexandria Journal of Medicine, 48(2), pp.167-173.

ALLEN, M. J. M., M.M, A., P.C, M., A.D, F. & O., T. 2013. - Self-reported practices in opioid management of chronic noncancer pain: A survey of canadian family physicians. Pain Research and Management, 18, 177-184.

ARDINER, C., M. GOTT, C. INGLETON, P. HUGHES, M. WINSLOW and M. I. BENNETT. 2012. Attitudes of health care professionals to opioid prescribing in end-of-life care: a qualitative focus group study. Journal of Pain & Symptom Management, 44(2), pp.206-14.

BIRO, E. 2013. Attitudes of Hungarian adults toward use of opioids in pain management. Orvosi Hetilap, 154(12), pp.455-463.

BLAKE, S., B. RUEL, C. SEAMARK and D. SEAMARK. 2007. - Experiences of patients requiring strong opioid drugs for chronic non-cancer pain: A patient-initiated study. British Journal of General Practice, 57(535), pp.101-109.

BUDKAEW, J. and B. CHUMWORATHAYI. 2013. Knowledge and attitudes toward palliative terminal cancer care among Thai generalists. Asian Pacific Journal of Cancer Prevention: Apjcp, 14(10), pp.6173-80.

CRANER, J. R., R. R. SKIPPER, W. P. GILLIAM, E. J. MORRISON and J. A. SPERRY. 2016. Patients’ perceptions of a chronic pain rehabilitation program: changing the conversation. Current Medical Research and Opinion, 32(5), pp.879-883.

EMANUEL, E. J., D. FAIRCLOUGH, B. C. CLARRIDGE, D. BLUM, E. BRUERA, W. C. PENLEY, L. E. SCHNIPPER and R. J. MAYER. 2000. - Attitudes and practices of U.S. oncologists regarding euthanasia and physician-assisted suicide. Annals of Internal Medicine, 133(7), pp.527-532.

KARANGES, E. A., A. SURAEV, N. ELIAS, A. R. MANOCH and I. S. MCGREGOR. 2018. Knowledge and attitudes of Australian general practitioners towards medicinal cannabis: A cross-sectional survey. BMJ Open, 8(7).

GALLAGHER, R., B. J.A, F. G, H. P and Y. W. 2003. - Attitudes and beliefs about the use of Cannabis for symptom control in a palliative population. Journal of Cannabis Therapeutics, 3(2), pp.41-50.

GUNNARSDOTTIR, S., H. S. DONOVAN, R. C. SERLIN, C. VOGE and S. WARD. 2002. Patient-related barriers to pain management: the Barriers Questionnaire II (BQ-II). Pain, 99(3), pp.385-96.

HANSEN, A. B., S. SKURTVEIT, P. C. BORCHGREVINK, O. DALE, P. R. ROMUNDSTAD, M. MAHIC and O. M. FREDHEIM. 2015. Consumption of and satisfaction with health care among opioid users with chronic non-malignant pain. Acta Anaesthesiologica Scandinavica, 59(10), pp.1355-66.

HUTCHINSON, K., A. M. MORELAND, C. W. A. C. DE, J. WEINMAN and R. HORNE. 2007. Exploring beliefs and practice of opioid prescribing for persistent non-cancer pain by general practitioners. European Journal of Pain, 11(1), pp.93-8.

JORANSON, D. E. and A. M. GILSON. 2001. Pharmacists' knowledge of and attitudes toward opioid pain medications in relation to federal and state policies. Journal of the American Pharmaceutical Association, 41(2), pp.213-20.

KARANGES, E. A., A. SURAEV, N. ELIAS, A. R. MANOCH and I. S. MCGREGOR. 2018. - Knowledge and attitudes of Australian general practitioners towards medicinal cannabis: A cross-sectional survey. BMJ Open, 8(7).

MORITA, T., M. MIYASHITA, M. SHIBAGAKI, K. HIRAI, T. ASHIYA, T. ISHIHARA, T. MATSUBARA, I. MIYOSHI, T. NAKAHO, N. NAKASHIMA, H. ONISHI, T. OZAWA, K. SUENAGA, T. TAJIMA, T. AKECHI and Y. UCHITOMI. 2006. Knowledge and beliefs about end-of-life care and the effects of specialized palliative care: a population-based survey in Japan. Journal of Pain & Symptom Management, 31(4), pp.306-16.

MYSTAKIDOU, K., E. TSILIKA, E. PARPA, P. GOGOU, P. THEODORAKIS and L. VLAHOS. 2010. Self-efficacy beliefs and levels of anxiety in advanced cancer patients. European Journal of Cancer Care, 19(2), pp.205-11.

NIEMI-MUROLA, L., N. J.T, K. E and P. R. 2007. - Medical undergraduate students' beliefs and attitudes toward pain - How do they mature? European Journal of Pain, 11(6), pp.700-706.

NUSEIR, K., M. KASSAB and B. ALMOMANI. 2016. Healthcare Providers&#x2019; Knowledge and Current Practice of Pain Assessment and Management: How Much Progress Have We Made? Pain Research and Management, 2016, pp1- 7.

NUSEIR, K., K. M and A. B. 2016. - Healthcare providers' knowledge and current practice of pain assessment and management: How much progress have we made? Pain Research and Management, (pagination).

OLIVER, D. P., W.-L. E, W. K, K. R.L, A. D.L, B. P.K, B. A and D. G. 2013. - Hospice caregivers' Experiences with pain management: "i'm not a doctor, and i don't know if i helped her go faster or slower". Journal of Pain and Symptom Management, 46(6), pp.846-858.

PALOS, G. R., T. R. MENDOZA, S. B. CANTOR, L. A. ADAY and C. S. CLEELAND. 2004. Perceptions of analgesic use and side effects: what the public values in pain management. Journal of Pain & Symptom Management, 28(5), pp.460-73.

PATEL, T., F. CHANG, H. T. MOHAMMED, L. RAMAN-WILMS, J. JURCIC, A. KHAN and B. SPROULE. 2016. - Knowledge, perceptions and attitudes toward chronic pain and its management: A cross-sectional survey of frontline pharmacists in Ontario, Canada. PLoS ONE, 11(6).

PUNTILLO, K. A., C. WHITE, A. B. MORRIS, S. T. PERDUE, J. STANIK-HUTT, C. L. THOMPSON and L. R. WILD. 2001. Patients' perceptions and responses to procedural pain: Results from Thunder Project II. American Journal of Critical Care, 10(4), pp.238-251.

ROBINSON, J. P., E. J. DANSIE, H. D. WILSON, S. RAPP and D. C. TURK. 2015. - Attitudes and Beliefs of Working and Work-Disabled People with Chronic Pain Prescribed Long-Term Opioids. Pain Medicine, 16(7), pp.1311-1324.

SHAW, S. and A. LEE. 2010. Student nurses' misconceptions of adults with chronic nonmalignant pain. Pain Management Nursing, 11(1), pp.2-14.

TALMI, Y. P., A. WALLER, M. BERCOVICI, Z. HOROWITZ, M. R. PFEFFER, A. ADUNSKI and J. KRONENBERG. 1997. - Pain experienced by patients with terminal head and neck carcinoma. Cancer, 80(6), pp.1117-1123.

VRANKEN, M. J. M., A. K. MANTEL‐TEEUWISSE, S. JÜNGER, L. RADBRUCH, W. SCHOLTEN, J. A. LISMAN, M. SUBATAITE and M. H. D. B. SCHUTJENS. 2017. Barriers to access to opioid medicines for patients with opioid dependence: a review of legislation and regulations in eleven central and eastern European countries. Addiction, 112(6), pp.1069-1076.

WEINSTEIN, S. M., L. F. LAUX, T. J.I, R. J. LORIMOR, J. C. S. HILL, D. M. THORPE and J. M. MERRILL. 2000. - Physicians' attitudes toward pain and the use of opioid analgesics: Results of a survey from the Texas cancer pain initiative. Southern Medical Journal, 93(5), pp.479-487.

WELSH, J., A. REID, J. GRAHAM, J. CURTO, K. MACLEOD and C. O'NEILL. 2005. Physicians' knowledge of transdermal fentanyl. Palliative Medicine, 19(1), pp.9-16.

YAMAMOTO, R., Y. KIZAWA, Y. NAKAZAWA and T. MORITA. 2013. The palliative care knowledge questionnaire for PEACE: reliability and validity of an instrument to measure palliative care knowledge among physicians. Journal of Palliative Medicine, 16(11), pp.1423-8.

ZEINAH, G. F., S. G. AL-KINDI and A. A. HASSAN. 2013. Attitudes of medical oncologists in Qatar toward palliative care. American Journal of Hospice & Palliative Medicine, 30(6), pp.548-551.

**Not published in English: n= 6**

GONG, S. W., J. Y. BANG, M. S. SEO, S. S. HYUN, H. J. KIM, M. LEE, H. H. YOU, J. K. HER, E. KIM and K. S. PARK. 2004. Knowledge and attitudes of oncology nurses toward cancer pain managements. Journal of Korean Academy of Adult Nursing, 16(1), pp.5-16.

KADA, O., R. LIKAR, W. PIPAM, M. MIKLAUTZ and H. JANIG. 2007. [Cancer pain from the nurses' perspective--findings from Austria]. Pflege Zeitschrift, 60(11),

SALVADÓ-HERNÁNDEZ, C., C. FUENTELSAZ-GALLEGO, C. ARCAY-VEIRA, C. LÓPEZ-MELÉNDEZ, T. VILLAR-ARNAL, N. CASAS-SEGALA and F. P. EN REPRESENTACIÓN DEL GRUPO DE INVESTIGACIÓN DEL PROYECTO. 2009. [Knowledge and attitudes on pain management by nurses in surgery and oncology in level III hospitals]. Enfermeria clinica, 19(6), pp.322-329.

SATO, Y. 2007. [An attitude survey on the medical use of narcotics for cancer pain relief]. Gan to kagaku ryoho. Cancer & chemotherapy, 34(13), pp.2267-2270.

TZEITLIN, T. and P. SHVARTZMAN. 2000. [Knowledge, attitudes and skills of family physicians in Israel with regard to chronic pain management in cancer]. Harefuah, 139(7-8), pp.252-5, 328.

WU, H.-B., M.-C. LEE, K.-H. LAI, S.-T. HO, W.-Z. SUN, J. O.-N. WONG and L.-P. GER. 2006. Physicians' knowledge about pharmacological management of cancer pain--with special reference on their prescribing responses to simulated patients with cancer pain. Acta anaesthesiologica Taiwanica: official journal of the Taiwan Society of Anesthesiologists, 44(2), pp.61-71.

**Not include adults: n= 5**

DE FREITAS, G. R., C. G. DE CASTRO, JR., S. M. CASTRO and I. HEINECK. 2014. Degree of knowledge of health care professionals about pain management and use of opioids in paediatrics. Pain Medicine, 15(5), pp.807-19.

FORWARD, S. P., T. L. BROWN and P. J. MCGRATH. 1996. Mothers' attitudes and behavior toward medicating children's pain. Pain, 67(2-3), pp.469-474.

FORTIER , M. A., WAHI, A., MAURER , E. L., TAN, E. T., SENDER, L. S. & KAIN, Z. N. 2012. - Attitudes regarding analgesic use and pain expression in parents of children with cancer. Journal of Pediatric Hematology/Oncology, 34, 257-262.

MANWORREN, R. C. 2000. Pediatric nurses' knowledge and attitudes survey regarding pain. Pediatric Nursing, 26, 610-4.

ROTH, M., D. DAVIES, S. FRIEBERT, D. WANG, M. KIM and S. ZELCER. 2013. Attitudes and Practices of Pediatric Oncologists Regarding Methadone Use in the Treatment of Cancer-related Pain: Results of a North American Survey. Journal of Pediatric Hematology Oncology, 35(2), pp.103-107.

**Not cross-sectional design: n= 5**

DE SILVA, B. S. S. and C. ROLLS. 2011. Attitudes, beliefs, and practices of Sri Lankan nurses toward cancer pain management: An ethnographic study. Nursing & Health Sciences, 13(4), pp.419-424.

HOWELL, D., L. BUTLER, L. VINCENT, J. WATT-WATSON and N. STEARNS. 2000. Influencing Nurses' Knowledge, Attitudes, and Practice in Cancer Pain Management. Cancer Nursing., 23(1), pp.55-63.

LOPEZ, L. R., H. C. CAMPS, P. KHOSRAVI-SHAHI, P. V. GUILLEM, M. A. CARRATO, J. GARCIA-FONCILLAS, H. J. J. CRUZ, V. P. GASCON, T. A. ANTON, E. DIAZ-RUBIO, M. FEYJOO SAUS and A. E. ARANDA. 2018. - Oncologist's knowledge and implementation of guidelines for breakthrough cancer pain in Spain: CONOCE study. Clinical and Translational Oncology, 20(5), pp.613-618.

LOPEZ DE MATURANA, A., MORAGO, V., SAN EMETERIO, E., GOROSTIZA, J. & ARRATE, A. O. 1993. Attitudes of general practitioners in Bizkaia, Spain, towards the terminally ill patient. Palliative Medicine, 7, 39-45.

PAICE, J. A., T. C and S. S. 1998. - Barriers to cancer pain relief: Fear of tolerance and addiction. Journal of Pain and Symptom Management, 16(1), pp.1-9.

**Same data were used in another study: n= 2**

ALNAJAR, M. K., M. W. DARAWAD, S. S. ALSHAHWAN and O. A. SAMARKANDI. 2017. Knowledge and Attitudes toward Cancer Pain Management among Nurses at Oncology Units. Journal of Cancer Education.

SALIM, N., N. AL-ATTYAT and M. TUFFAHA. 2017. Knowledge and Attitude of Oncology Nurses toward Cancer Pain Management: A Review. Arch Med, 9, p2.

**Full-text article not found: n= 1**

JUBELIRER, S. J., C. S. WARREN, R. RECTOR, G. KUHN, S. WALTON and C. ZINN. 1998. Attitudes about cancer pain: a survey of 727 health care professionals in West Virginia. The West Virginia medical journal, 94(4), pp.192-194.

**“Letter to the editor”** **(not enough information were stated): n= 1**

LAMBERT, K., S. OXBERRY, C. W. HULME, K. SAHARIA, A. S. RIGBY and M. J. JOHNSON. 2007. Knowledge and attitudes to opioids in palliative care patients. Palliative Medicine, 21(8), pp.721-722.

##### Appendix 5 Search strategy for 6 databases:

**CINAHL search screen strategy**

| **Search ID #** | **Search Terms** | **Results** |
| --- | --- | --- |

| S17 | (adult*) AND (S3 AND S7 AND S10 AND S15 AND S16) | 243 |
| --- | --- | --- |
| S16 | adult* | 688,224 |
| S15 | (morphine* OR opium* OR opioid* OR opiate* OR buprenorphine* OR codeine* OR diamorphine* OR dihydrocodeine* OR alfentanil* OR fentanyl* OR oxycodone* OR hydromorphine*) AND (S11 OR S12 OR S13 OR S14) | 25,776 |
| S14 | morphine* OR opium* OR opioid* OR opiate* OR buprenorphine* OR codeine* OR diamorphine* OR dihydrocodeine* OR alfentanil* OR fentanyl* OR oxycodone* OR hydromorphine* | 25,776 |
| S13 | (MH "Narcotics+") | 21,162 |
| S12 | (MH "Morphine+") | 8,891 |
| S11 | (MH "Analgesics+") | 27,283 |
| S10 | (cancer* OR tumor* OR carcinoma* OR leuk?emia* OR metasta* OR lymphoma* OR melanoma* OR oncolog*) AND (S8 OR S9) | 270,901 |
| S9 | cancer* OR tumor* OR carcinoma* OR leuk?emia* OR metasta* OR lymphoma* OR melanoma* OR oncolog* | 270,901 |
| S8 | (MH "Neoplasms+") | 246,112 |
| S7 | (pain*) AND (S4 OR S5 OR S6) | 173,267 |
| S6 | pain* | 173,267 |
| S5 | (MM "Pain Management") | 1,101 |
| S4 | (MH "Pain+") | 119,800 |
| S3 | (standpoint* OR expectation* OR preference* OR need* OR satisfaction* OR interaction*) AND (S1 OR S2) | 496,987 |
| S2 | standpoint* OR expectation* OR preference* OR need* OR satisfaction* OR interaction* | 496,987 |
| S1 | view* OR opinion* OR attitude* OR concern* OR belief* OR feeling* OR idea* OR perception* OR perspective* OR experience* OR knowledge* OR perceive* | 715,761 |

**PsycINFO Search Strategy**

|  |  |  |  |  |
| --- | --- | --- | --- | --- |
| 1. adult*.mp. [mp=title, abstract, heading word, table of contents, key concepts, original title, tests & measures] | 462957 | |  |  |
| 1. (view* or opinion* or attitude* or concern* or belief* or feeling* or idea* or perception* or perspective* or experience* or knowledge* or perceive* or standpoint* or expectation* or preference* or need* or satisfaction* or interaction*).af. | | 3153834 |  |  |
| 1. exp PAIN/ | | 53046 |  |  |
| 1. exp Pain Management/ | | 8721 |  |  |
| 1. pain*.mp. [mp=title, abstract, heading word, table of contents, key concepts, original title, tests & measures] | | 107982 |  |  |
| 1. 3 or 4 or 5 | | 118006 |  |  |
| 1. exp NEOPLASMS/ | | 47152 |  |  |
| 1. (cancer* or tumor* or carcinoma* or leuk?emia* or metasta* or lymphoma* or melanoma* or oncolog*).af. | | 249091 |  |  |
| 1. 7 or 8 | | 249742 |  |  |
| 1. exp ANALGESIC DRUGS/ | | 18265 |  |  |
| 1. exp MORPHINE/ | | 6650 |  |  |
| 1. exp NARCOTIC DRUGS/ | | 27313 |  |  |
| 1. (morphine* or opium* or opioid* or opiate* or buprenorphine* or codeine* or diamorphine* or dihydrocodeine* or alfentanil* or fentanyl* or oxycodone* or hydromorphine*).af. | | 94933 |  |  |
| 1. 10 or 11 or 12 or 13 | | 103192 |  |  |
| 1. 1 and 2 and 6 and 9 and 14 | | 647 |  |  |
|  | |  |  |  |

**EMBASE search strategy**

|  | (view* or opinion* or attitude* or concern* or belief* or feeling* or dea* or perception* or perspective* or experience* or knowledge* or perceive* or standpoint* or expectation* or preference* or need* or satisfaction* or interaction*).af. | | 7062565 | | Advanced | [Display Results](http://0-ovidsp.uk.ovid.com.wam.leeds.ac.uk/sp-3.31.1b/ovidweb.cgi?&S=OOFBPDMCGMHFPJEMFNEKBGDGPOCJAA00&SELECT=S.sh%7c&R=1&Process+Action=display)  [More](http://0-ovidsp.uk.ovid.com.wam.leeds.ac.uk/sp-3.31.1b/ovidweb.cgi) | [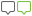](http://0-ovidsp.uk.ovid.com.wam.leeds.ac.uk/sp-3.31.1b/ovidweb.cgi?&S=OOFBPDMCGMHFPJEMFNEKBGDGPOCJAA00&R=1&Search+Annotations+Options=SA) |
| --- | --- | --- | --- | --- | --- | --- | --- |
| 1. exp pain/ | | 1012089 | |  |  |  |  |
| 1. pain*.mp. [mp=title, abstract, heading word, drug trade name, original title, device manufacturer, drug manufacturer, device trade name, keyword, floating subheading word, candidate term word] | | 1015525 | |  |  |  |  |
| 1. 2 or 3 | | 1254670 | |  |  |  |  |
| 1. exp neoplasm/ | | 3087277 | |  |  |  |  |
| 1. (cancer* or tumor* or carcinoma* or leuk?emia* or metasta* or lymphoma* or melanoma* or oncolog*).af. | | 4280648 | |  |  |  |  |
| 1. 5 or 6 | | 4482350 | |  |  |  |  |
| 1. exp analgesic agent/ | | 598302 | |  |  |  |  |
| 1. exp morphine/ | | 60583 | |  |  |  |  |
| 1. exp narcotic agent/ | | 172089 | |  |  |  |  |
| 1. (morphine* or opium* or opioid* or opiate* or buprenorphine* or codeine* or diamorphine* or dihydrocodeine* or alfentanil* or fentanyl* or oxycodone* or hydromorphine*).af. | | 218527 | |  |  |  |  |
| 1. 8 or 9 or 10 or 11 | | 639968 | |  |  |  |  |
| 1. adult*.mp. [mp=title, abstract, heading word, drug trade name, original title, device manufacturer, drug manufacturer, device trade name, keyword, floating subheading word, candidate term word] | | 5322545 | |  |  |  |  |
| 1. 1 and 4 and 7 and 12 and 13 | | 7241 | |  |  |  |  |
| 1. limit 14 to (human and male and female and english language and embase and english and article and journal and adult <18 to 64 years>) | | 2947 | |  |  |  |  |

**Cochrane Search Strategy**

**ID** **Search Teams Results**

#1 view* or opinion* or attitude* or concern* or belief* or feeling* or idea* or perception* or perspective* or experience* or knowledge* or perceive* or standpoint*or expectation* or preference* or need* or satisfaction* or interaction* 359578

#2 pain* 139399

#3 MeSH descriptor: [Pain] explode all trees 45245

#4 MeSH descriptor: [Pain Management] explode all trees 2999

#5 #2 or #3 or #4 145313

#6 MeSH descriptor: [Neoplasms] explode all trees 78711

#7 cancer* or tumor* or carcinoma* or leuk?emia* or metasta* or lymphoma* or melanoma* or oncolog* 183110

#8 #6 or #7 191862

#9 MeSH descriptor: [Analgesics] explode all trees 20074

#10 MeSH descriptor: [Morphine] explode all trees 4599

#11 MeSH descriptor: [Narcotics] explode all trees 7750

#12 morphine* or opium* or opioid* or opiate* or buprenorphine* or codeine* or diamorphine* or dihydrocodeine* or alfentanil* or fentanyl* or oxycodone* or hydromorphine* 37382

#13 #9 or #10 or #11 or #12 48210

#14 adult* 532482

#15 #1 and #5 and #8 and #13 and #14 1090

**MEDLINE Search strategy**

**Search terms** **Results**

1. (view* or opinion* or attitude* or concern* or belief* or feeling* or idea* or perception* or perspective* or experience* or knowledge* or perceive* or standpoint*or expectation* or preference* or need* or satisfaction* or interaction*).af.

(4960401)

1. exp PAIN/ (360610)
2. exp pain management/ (28230)
3. pain* (608567)
4. 2 or 3 or 4 (683477)
5. Exp neoplasms/ (3061130)
6. (cancer* or tumor* or carcinoma* or leuk?emia* or metasta* or lymphoma* or melanoma* or oncolog*).af. (3309961)
7. 6 or 7 (3925212)
8. exp Analgesics/ (495873)
9. exp morphine/ (36852)
10. exp narcotics/ (112857)
11. (morphine* or opium* or opioid* or opiate* or buprenorphine* or codeine* or diamorphine* or dihydrocodeine* or alfentanil* or fentanyl* or oxycodone* or hydromorphine*).af. (149692)
12. 9 or 10 or 11 or 12 (544246)
13. adult* (5229241)
14. 1 and 5 and 8 and 13 and 14 (1864)

**Web of Science Search strategy**

| Search History | | | | | |
| --- | --- | --- | --- | --- | --- |
| **Set** | **Results** | **Save History / Create AlertOpen Saved History** | **Edit Sets** | **Combine Sets**  ** AND   OR**  **Combine** | **Delete Sets**  **Select All  Delete** |
|  | | | | | |
| # 40 | [**2,598**](http://0-apps.webofknowledge.com.wam.leeds.ac.uk/summary.do?product=WOS&doc=1&qid=44&SID=E6DF347xpvyl1VP4woH&search_mode=CombineSearches&update_back2search_link_param=yes) | #39 AND #29 AND #26 AND #12 AND #5  *Indexes=SCI-EXPANDED, SSCI, A&HCI, CPCI-S, CPCI-SSH, ESCI Timespan=All years* | [Edit](http://0-apps.webofknowledge.com.wam.leeds.ac.uk/WOS_AdvancedSearch_input.do?product=WOS&SID=E6DF347xpvyl1VP4woH&search_mode=AdvancedSearch&replaceSetId=40&editState=init) |  |  |
|  | | | | | |
| # 39 | [**3,690,512**](http://0-apps.webofknowledge.com.wam.leeds.ac.uk/summary.do?product=WOS&doc=1&qid=43&SID=E6DF347xpvyl1VP4woH&search_mode=CombineSearches&update_back2search_link_param=yes) | #38 OR #37 OR #36 OR #35 OR #34 OR #33 OR #32 OR #31 OR #30  *Indexes=SCI-EXPANDED, SSCI, A&HCI, CPCI-S, CPCI-SSH, ESCI Timespan=All years* | [Edit](http://0-apps.webofknowledge.com.wam.leeds.ac.uk/WOS_AdvancedSearch_input.do?product=WOS&SID=E6DF347xpvyl1VP4woH&search_mode=AdvancedSearch&replaceSetId=39&editState=init) |  |  |
|  | | | | | |
| # 38 | [**164,157**](http://0-apps.webofknowledge.com.wam.leeds.ac.uk/summary.do?product=WOS&doc=1&qid=42&SID=E6DF347xpvyl1VP4woH&search_mode=GeneralSearch&update_back2search_link_param=yes) | **TOPIC:** (oncolog*)  *Indexes=SCI-EXPANDED, SSCI, A&HCI, CPCI-S, CPCI-SSH, ESCI Timespan=All years* | [Edit](http://0-apps.webofknowledge.com.wam.leeds.ac.uk/WOS_AdvancedSearch_input.do?product=WOS&SID=E6DF347xpvyl1VP4woH&search_mode=AdvancedSearch&replaceSetId=38&editState=init) |  |  |
|  | | | | | |
| # 37 | [**148,960**](http://0-apps.webofknowledge.com.wam.leeds.ac.uk/summary.do?product=WOS&doc=1&qid=41&SID=E6DF347xpvyl1VP4woH&search_mode=GeneralSearch&update_back2search_link_param=yes) | **TOPIC:** (melanoma*)  *Indexes=SCI-EXPANDED, SSCI, A&HCI, CPCI-S, CPCI-SSH, ESCI Timespan=All years* | [Edit](http://0-apps.webofknowledge.com.wam.leeds.ac.uk/WOS_AdvancedSearch_input.do?product=WOS&SID=E6DF347xpvyl1VP4woH&search_mode=AdvancedSearch&replaceSetId=37&editState=init) |  |  |
|  | | | | | |
| # 36 | [**208,282**](http://0-apps.webofknowledge.com.wam.leeds.ac.uk/summary.do?product=WOS&doc=1&qid=40&SID=E6DF347xpvyl1VP4woH&search_mode=GeneralSearch&update_back2search_link_param=yes) | **TOPIC:** (lymphoma*)  *Indexes=SCI-EXPANDED, SSCI, A&HCI, CPCI-S, CPCI-SSH, ESCI Timespan=All years* | [Edit](http://0-apps.webofknowledge.com.wam.leeds.ac.uk/WOS_AdvancedSearch_input.do?product=WOS&SID=E6DF347xpvyl1VP4woH&search_mode=AdvancedSearch&replaceSetId=36&editState=init) |  |  |
|  | | | | | |
| # 35 | [**498,998**](http://0-apps.webofknowledge.com.wam.leeds.ac.uk/summary.do?product=WOS&doc=1&qid=39&SID=E6DF347xpvyl1VP4woH&search_mode=GeneralSearch&update_back2search_link_param=yes) | **TOPIC:** (malignan*)  *Indexes=SCI-EXPANDED, SSCI, A&HCI, CPCI-S, CPCI-SSH, ESCI Timespan=All years* | [Edit](http://0-apps.webofknowledge.com.wam.leeds.ac.uk/WOS_AdvancedSearch_input.do?product=WOS&SID=E6DF347xpvyl1VP4woH&search_mode=AdvancedSearch&replaceSetId=35&editState=init) |  |  |
|  | | | | | |
| # 34 | [**528,796**](http://0-apps.webofknowledge.com.wam.leeds.ac.uk/summary.do?product=WOS&doc=1&qid=38&SID=E6DF347xpvyl1VP4woH&search_mode=GeneralSearch&update_back2search_link_param=yes) | **TOPIC:** (metasta*)  *Indexes=SCI-EXPANDED, SSCI, A&HCI, CPCI-S, CPCI-SSH, ESCI Timespan=All years* | [Edit](http://0-apps.webofknowledge.com.wam.leeds.ac.uk/WOS_AdvancedSearch_input.do?product=WOS&SID=E6DF347xpvyl1VP4woH&search_mode=AdvancedSearch&replaceSetId=34&editState=init) |  |  |
|  | | | | | |
| # 33 | [**29,097**](http://0-apps.webofknowledge.com.wam.leeds.ac.uk/summary.do?product=WOS&doc=1&qid=37&SID=E6DF347xpvyl1VP4woH&search_mode=GeneralSearch&update_back2search_link_param=yes) | **TOPIC:** (leuk?emia*)  *Indexes=SCI-EXPANDED, SSCI, A&HCI, CPCI-S, CPCI-SSH, ESCI Timespan=All years* | [Edit](http://0-apps.webofknowledge.com.wam.leeds.ac.uk/WOS_AdvancedSearch_input.do?product=WOS&SID=E6DF347xpvyl1VP4woH&search_mode=AdvancedSearch&replaceSetId=33&editState=init) |  |  |
|  | | | | | |
| # 32 | [**841,643**](http://0-apps.webofknowledge.com.wam.leeds.ac.uk/summary.do?product=WOS&doc=1&qid=36&SID=E6DF347xpvyl1VP4woH&search_mode=GeneralSearch&update_back2search_link_param=yes) | **TOPIC:** (carcinoma*)  *Indexes=SCI-EXPANDED, SSCI, A&HCI, CPCI-S, CPCI-SSH, ESCI Timespan=All years* | [Edit](http://0-apps.webofknowledge.com.wam.leeds.ac.uk/WOS_AdvancedSearch_input.do?product=WOS&SID=E6DF347xpvyl1VP4woH&search_mode=AdvancedSearch&replaceSetId=32&editState=init) |  |  |
|  | | | | | |
| # 31 | [**1,600,248**](http://0-apps.webofknowledge.com.wam.leeds.ac.uk/summary.do?product=WOS&doc=1&qid=35&SID=E6DF347xpvyl1VP4woH&search_mode=GeneralSearch&update_back2search_link_param=yes) | **TOPIC:** (tumo*)  *Indexes=SCI-EXPANDED, SSCI, A&HCI, CPCI-S, CPCI-SSH, ESCI Timespan=All years* | [Edit](http://0-apps.webofknowledge.com.wam.leeds.ac.uk/WOS_AdvancedSearch_input.do?product=WOS&SID=E6DF347xpvyl1VP4woH&search_mode=AdvancedSearch&replaceSetId=31&editState=init) |  |  |
|  | | | | | |
| # 30 | [**2,150,502**](http://0-apps.webofknowledge.com.wam.leeds.ac.uk/summary.do?product=WOS&doc=1&qid=34&SID=E6DF347xpvyl1VP4woH&search_mode=GeneralSearch&update_back2search_link_param=yes) | **TOPIC:** (Cancer*)  *Indexes=SCI-EXPANDED, SSCI, A&HCI, CPCI-S, CPCI-SSH, ESCI Timespan=All years* | [Edit](http://0-apps.webofknowledge.com.wam.leeds.ac.uk/WOS_AdvancedSearch_input.do?product=WOS&SID=E6DF347xpvyl1VP4woH&search_mode=AdvancedSearch&replaceSetId=30&editState=init) |  |  |
|  | | | | | |
| # 29 | [**658,408**](http://0-apps.webofknowledge.com.wam.leeds.ac.uk/summary.do?product=WOS&doc=1&qid=33&SID=E6DF347xpvyl1VP4woH&search_mode=CombineSearches&update_back2search_link_param=yes) | #28 OR #27  *Indexes=SCI-EXPANDED, SSCI, A&HCI, CPCI-S, CPCI-SSH, ESCI Timespan=All years* | [Edit](http://0-apps.webofknowledge.com.wam.leeds.ac.uk/WOS_AdvancedSearch_input.do?product=WOS&SID=E6DF347xpvyl1VP4woH&search_mode=AdvancedSearch&replaceSetId=29&editState=init) |  |  |
|  | | | | | |
| # 28 | [**92,793**](http://0-apps.webofknowledge.com.wam.leeds.ac.uk/summary.do?product=WOS&doc=1&qid=32&SID=E6DF347xpvyl1VP4woH&search_mode=GeneralSearch&update_back2search_link_param=yes) | **TOPIC:** (pain management*)  *Indexes=SCI-EXPANDED, SSCI, A&HCI, CPCI-S, CPCI-SSH, ESCI Timespan=All years* | [Edit](http://0-apps.webofknowledge.com.wam.leeds.ac.uk/WOS_AdvancedSearch_input.do?product=WOS&SID=E6DF347xpvyl1VP4woH&search_mode=AdvancedSearch&replaceSetId=28&editState=init) |  |  |
|  | | | | | |
| # 27 | [**658,408**](http://0-apps.webofknowledge.com.wam.leeds.ac.uk/summary.do?product=WOS&doc=1&qid=31&SID=E6DF347xpvyl1VP4woH&search_mode=GeneralSearch&update_back2search_link_param=yes) | **TOPIC:** (pain*)  *Indexes=SCI-EXPANDED, SSCI, A&HCI, CPCI-S, CPCI-SSH, ESCI Timespan=All years* | [Edit](http://0-apps.webofknowledge.com.wam.leeds.ac.uk/WOS_AdvancedSearch_input.do?product=WOS&SID=E6DF347xpvyl1VP4woH&search_mode=AdvancedSearch&replaceSetId=27&editState=init) |  |  |
|  | | | | | |
| # 26 | [**6,073,162**](http://0-apps.webofknowledge.com.wam.leeds.ac.uk/summary.do?product=WOS&doc=1&qid=30&SID=E6DF347xpvyl1VP4woH&search_mode=CombineSearches&update_back2search_link_param=yes) | #25 OR #24 OR #23 OR #22 OR #21 OR #20 OR #19 OR #18 OR #17 OR #16 OR #15 OR #14 OR #13  *Indexes=SCI-EXPANDED, SSCI, A&HCI, CPCI-S, CPCI-SSH, ESCI Timespan=All years* | [Edit](http://0-apps.webofknowledge.com.wam.leeds.ac.uk/WOS_AdvancedSearch_input.do?product=WOS&SID=E6DF347xpvyl1VP4woH&search_mode=AdvancedSearch&replaceSetId=26&editState=init) |  |  |
|  | | | | | |
| # 25 | [**24,716**](http://0-apps.webofknowledge.com.wam.leeds.ac.uk/summary.do?product=WOS&doc=1&qid=29&SID=E6DF347xpvyl1VP4woH&search_mode=GeneralSearch&update_back2search_link_param=yes) | **TOPIC:** (standpoint*)  *Indexes=SCI-EXPANDED, SSCI, A&HCI, CPCI-S, CPCI-SSH, ESCI Timespan=All years* | [Edit](http://0-apps.webofknowledge.com.wam.leeds.ac.uk/WOS_AdvancedSearch_input.do?product=WOS&SID=E6DF347xpvyl1VP4woH&search_mode=AdvancedSearch&replaceSetId=25&editState=init) |  |  |
|  | | | | | |
| # 24 | [**319,377**](http://0-apps.webofknowledge.com.wam.leeds.ac.uk/summary.do?product=WOS&doc=1&qid=28&SID=E6DF347xpvyl1VP4woH&search_mode=GeneralSearch&update_back2search_link_param=yes) | **TOPIC:** (perceive*)  *Indexes=SCI-EXPANDED, SSCI, A&HCI, CPCI-S, CPCI-SSH, ESCI Timespan=All years* | [Edit](http://0-apps.webofknowledge.com.wam.leeds.ac.uk/WOS_AdvancedSearch_input.do?product=WOS&SID=E6DF347xpvyl1VP4woH&search_mode=AdvancedSearch&replaceSetId=24&editState=init) |  |  |
|  | | | | | |
| # 23 | [**1,172,275**](http://0-apps.webofknowledge.com.wam.leeds.ac.uk/summary.do?product=WOS&doc=1&qid=26&SID=E6DF347xpvyl1VP4woH&search_mode=GeneralSearch&update_back2search_link_param=yes) | **TOPIC:** (knowledge*)  *Indexes=SCI-EXPANDED, SSCI, A&HCI, CPCI-S, CPCI-SSH, ESCI Timespan=All years* | [Edit](http://0-apps.webofknowledge.com.wam.leeds.ac.uk/WOS_AdvancedSearch_input.do?product=WOS&SID=E6DF347xpvyl1VP4woH&search_mode=AdvancedSearch&replaceSetId=23&editState=init) |  |  |
|  | | | | | |
| # 22 | [**1,481,238**](http://0-apps.webofknowledge.com.wam.leeds.ac.uk/summary.do?product=WOS&doc=1&qid=25&SID=E6DF347xpvyl1VP4woH&search_mode=GeneralSearch&update_back2search_link_param=yes) | **TOPIC:** (experience*)  *Indexes=SCI-EXPANDED, SSCI, A&HCI, CPCI-S, CPCI-SSH, ESCI Timespan=All years* | [Edit](http://0-apps.webofknowledge.com.wam.leeds.ac.uk/WOS_AdvancedSearch_input.do?product=WOS&SID=E6DF347xpvyl1VP4woH&search_mode=AdvancedSearch&replaceSetId=22&editState=init) |  |  |
|  | | | | | |
| # 21 | [**752,315**](http://0-apps.webofknowledge.com.wam.leeds.ac.uk/summary.do?product=WOS&doc=1&qid=24&SID=E6DF347xpvyl1VP4woH&search_mode=GeneralSearch&update_back2search_link_param=yes) | **TOPIC:** (perspective*)  *Indexes=SCI-EXPANDED, SSCI, A&HCI, CPCI-S, CPCI-SSH, ESCI Timespan=All years* | [Edit](http://0-apps.webofknowledge.com.wam.leeds.ac.uk/WOS_AdvancedSearch_input.do?product=WOS&SID=E6DF347xpvyl1VP4woH&search_mode=AdvancedSearch&replaceSetId=21&editState=init) |  |  |
|  | | | | | |
| # 20 | [**496,299**](http://0-apps.webofknowledge.com.wam.leeds.ac.uk/summary.do?product=WOS&doc=1&qid=23&SID=E6DF347xpvyl1VP4woH&search_mode=GeneralSearch&update_back2search_link_param=yes) | **TOPIC:** (perception*)  *Indexes=SCI-EXPANDED, SSCI, A&HCI, CPCI-S, CPCI-SSH, ESCI Timespan=All years* | [Edit](http://0-apps.webofknowledge.com.wam.leeds.ac.uk/WOS_AdvancedSearch_input.do?product=WOS&SID=E6DF347xpvyl1VP4woH&search_mode=AdvancedSearch&replaceSetId=20&editState=init) |  |  |
|  | | | | | |
| # 19 | [**759,535**](http://0-apps.webofknowledge.com.wam.leeds.ac.uk/summary.do?product=WOS&doc=1&qid=22&SID=E6DF347xpvyl1VP4woH&search_mode=GeneralSearch&update_back2search_link_param=yes) | **TOPIC:** (idea*)  *Indexes=SCI-EXPANDED, SSCI, A&HCI, CPCI-S, CPCI-SSH, ESCI Timespan=All years* | [Edit](http://0-apps.webofknowledge.com.wam.leeds.ac.uk/WOS_AdvancedSearch_input.do?product=WOS&SID=E6DF347xpvyl1VP4woH&search_mode=AdvancedSearch&replaceSetId=19&editState=init) |  |  |
|  | | | | | |
| # 18 | [**78,344**](http://0-apps.webofknowledge.com.wam.leeds.ac.uk/summary.do?product=WOS&doc=1&qid=21&SID=E6DF347xpvyl1VP4woH&search_mode=GeneralSearch&update_back2search_link_param=yes) | **TOPIC:** (feeling*)  *Indexes=SCI-EXPANDED, SSCI, A&HCI, CPCI-S, CPCI-SSH, ESCI Timespan=All years* | [Edit](http://0-apps.webofknowledge.com.wam.leeds.ac.uk/WOS_AdvancedSearch_input.do?product=WOS&SID=E6DF347xpvyl1VP4woH&search_mode=AdvancedSearch&replaceSetId=18&editState=init) |  |  |
|  | | | | | |
| # 17 | [**170,183**](http://0-apps.webofknowledge.com.wam.leeds.ac.uk/summary.do?product=WOS&doc=1&qid=20&SID=E6DF347xpvyl1VP4woH&search_mode=GeneralSearch&update_back2search_link_param=yes) | **TOPIC:** (belief*)  *Indexes=SCI-EXPANDED, SSCI, A&HCI, CPCI-S, CPCI-SSH, ESCI Timespan=All years* | [Edit](http://0-apps.webofknowledge.com.wam.leeds.ac.uk/WOS_AdvancedSearch_input.do?product=WOS&SID=E6DF347xpvyl1VP4woH&search_mode=AdvancedSearch&replaceSetId=17&editState=init) |  |  |
|  | | | | | |
| # 16 | [**977,886**](http://0-apps.webofknowledge.com.wam.leeds.ac.uk/summary.do?product=WOS&doc=1&qid=19&SID=E6DF347xpvyl1VP4woH&search_mode=GeneralSearch&update_back2search_link_param=yes) | **TOPIC:** (concern*)  *Indexes=SCI-EXPANDED, SSCI, A&HCI, CPCI-S, CPCI-SSH, ESCI Timespan=All years* | [Edit](http://0-apps.webofknowledge.com.wam.leeds.ac.uk/WOS_AdvancedSearch_input.do?product=WOS&SID=E6DF347xpvyl1VP4woH&search_mode=AdvancedSearch&replaceSetId=16&editState=init) |  |  |
|  | | | | | |
| # 15 | [**314,379**](http://0-apps.webofknowledge.com.wam.leeds.ac.uk/summary.do?product=WOS&doc=1&qid=18&SID=E6DF347xpvyl1VP4woH&search_mode=GeneralSearch&update_back2search_link_param=yes) | **TOPIC:** (attitude*)  *Indexes=SCI-EXPANDED, SSCI, A&HCI, CPCI-S, CPCI-SSH, ESCI Timespan=All years* | [Edit](http://0-apps.webofknowledge.com.wam.leeds.ac.uk/WOS_AdvancedSearch_input.do?product=WOS&SID=E6DF347xpvyl1VP4woH&search_mode=AdvancedSearch&replaceSetId=15&editState=init) |  |  |
|  | | | | | |
| # 14 | [**147,361**](http://0-apps.webofknowledge.com.wam.leeds.ac.uk/summary.do?product=WOS&doc=1&qid=17&SID=E6DF347xpvyl1VP4woH&search_mode=GeneralSearch&update_back2search_link_param=yes) | **TOPIC:** (opinion*)  *Indexes=SCI-EXPANDED, SSCI, A&HCI, CPCI-S, CPCI-SSH, ESCI Timespan=All years* | [Edit](http://0-apps.webofknowledge.com.wam.leeds.ac.uk/WOS_AdvancedSearch_input.do?product=WOS&SID=E6DF347xpvyl1VP4woH&search_mode=AdvancedSearch&replaceSetId=14&editState=init) |  |  |
|  | | | | | |
| # 13 | [**985,752**](http://0-apps.webofknowledge.com.wam.leeds.ac.uk/summary.do?product=WOS&doc=1&qid=16&SID=E6DF347xpvyl1VP4woH&search_mode=GeneralSearch&update_back2search_link_param=yes) | **TOPIC:** (View*)  *Indexes=SCI-EXPANDED, SSCI, A&HCI, CPCI-S, CPCI-SSH, ESCI Timespan=All years* | [Edit](http://0-apps.webofknowledge.com.wam.leeds.ac.uk/WOS_AdvancedSearch_input.do?product=WOS&SID=E6DF347xpvyl1VP4woH&search_mode=AdvancedSearch&replaceSetId=13&editState=init) |  |  |
|  | | | | | |
| # 12 | [**152,087**](http://0-apps.webofknowledge.com.wam.leeds.ac.uk/summary.do?product=WOS&doc=1&qid=15&SID=E6DF347xpvyl1VP4woH&search_mode=CombineSearches&update_back2search_link_param=yes) | #11 OR #10 OR #9 OR #8 OR #7 OR #6  *Indexes=SCI-EXPANDED, SSCI, A&HCI, CPCI-S, CPCI-SSH, ESCI Timespan=All years* | [Edit](http://0-apps.webofknowledge.com.wam.leeds.ac.uk/WOS_AdvancedSearch_input.do?product=WOS&SID=E6DF347xpvyl1VP4woH&search_mode=AdvancedSearch&replaceSetId=12&editState=init) |  |  |
|  | | | | | |
| # 11 | [**27,343**](http://0-apps.webofknowledge.com.wam.leeds.ac.uk/summary.do?product=WOS&doc=1&qid=14&SID=E6DF347xpvyl1VP4woH&search_mode=GeneralSearch&update_back2search_link_param=yes) | **TOPIC:** (opiate*)  *Indexes=SCI-EXPANDED, SSCI, A&HCI, CPCI-S, CPCI-SSH, ESCI Timespan=All years* | [Edit](http://0-apps.webofknowledge.com.wam.leeds.ac.uk/WOS_AdvancedSearch_input.do?product=WOS&SID=E6DF347xpvyl1VP4woH&search_mode=AdvancedSearch&replaceSetId=11&editState=init) |  |  |
|  | | | | | |
| # 10 | [**1**](http://0-apps.webofknowledge.com.wam.leeds.ac.uk/summary.do?product=WOS&doc=1&qid=13&SID=E6DF347xpvyl1VP4woH&search_mode=GeneralSearch&update_back2search_link_param=yes) | **TOPIC:** (poium*)  *Indexes=SCI-EXPANDED, SSCI, A&HCI, CPCI-S, CPCI-SSH, ESCI Timespan=All years* | [Edit](http://0-apps.webofknowledge.com.wam.leeds.ac.uk/WOS_AdvancedSearch_input.do?product=WOS&SID=E6DF347xpvyl1VP4woH&search_mode=AdvancedSearch&replaceSetId=10&editState=init) |  |  |
|  | | | | | |
| # 9 | [**60,482**](http://0-apps.webofknowledge.com.wam.leeds.ac.uk/summary.do?product=WOS&doc=1&qid=12&SID=E6DF347xpvyl1VP4woH&search_mode=GeneralSearch&update_back2search_link_param=yes) | **TOPIC:** (morphine*)  *Indexes=SCI-EXPANDED, SSCI, A&HCI, CPCI-S, CPCI-SSH, ESCI Timespan=All years* | [Edit](http://0-apps.webofknowledge.com.wam.leeds.ac.uk/WOS_AdvancedSearch_input.do?product=WOS&SID=E6DF347xpvyl1VP4woH&search_mode=AdvancedSearch&replaceSetId=9&editState=init) |  |  |
|  | | | | | |
| # 8 | [**60,482**](http://0-apps.webofknowledge.com.wam.leeds.ac.uk/summary.do?product=WOS&doc=1&qid=10&SID=E6DF347xpvyl1VP4woH&search_mode=GeneralSearch&update_back2search_link_param=yes) | **TOPIC:** (morphine*)  *Indexes=SCI-EXPANDED, SSCI, A&HCI, CPCI-S, CPCI-SSH, ESCI Timespan=All years* | [Edit](http://0-apps.webofknowledge.com.wam.leeds.ac.uk/WOS_AdvancedSearch_input.do?product=WOS&SID=E6DF347xpvyl1VP4woH&search_mode=AdvancedSearch&replaceSetId=8&editState=init) |  |  |
|  | | | | | |
| # 7 | [**86,329**](http://0-apps.webofknowledge.com.wam.leeds.ac.uk/summary.do?product=WOS&doc=1&qid=9&SID=E6DF347xpvyl1VP4woH&search_mode=GeneralSearch&update_back2search_link_param=yes) | **TOPIC:** (opioid*)  *Indexes=SCI-EXPANDED, SSCI, A&HCI, CPCI-S, CPCI-SSH, ESCI Timespan=All years* | [Edit](http://0-apps.webofknowledge.com.wam.leeds.ac.uk/WOS_AdvancedSearch_input.do?product=WOS&SID=E6DF347xpvyl1VP4woH&search_mode=AdvancedSearch&replaceSetId=7&editState=init) |  |  |
|  | | | | | |
| # 6 | [**24,522**](http://0-apps.webofknowledge.com.wam.leeds.ac.uk/summary.do?product=WOS&doc=1&qid=8&SID=E6DF347xpvyl1VP4woH&search_mode=GeneralSearch&update_back2search_link_param=yes) | **TOPIC:** (Analgesics*)  *Indexes=SCI-EXPANDED, SSCI, A&HCI, CPCI-S, CPCI-SSH, ESCI Timespan=All years* | [Edit](http://0-apps.webofknowledge.com.wam.leeds.ac.uk/WOS_AdvancedSearch_input.do?product=WOS&SID=E6DF347xpvyl1VP4woH&search_mode=AdvancedSearch&replaceSetId=6&editState=init) |  |  |
|  | | | | | |
| # 5 | [**7,178,014**](http://0-apps.webofknowledge.com.wam.leeds.ac.uk/summary.do?product=WOS&doc=1&qid=7&SID=E6DF347xpvyl1VP4woH&search_mode=CombineSearches&update_back2search_link_param=yes) | #4 OR #3 OR #2 OR #1  *Indexes=SCI-EXPANDED, SSCI, A&HCI, CPCI-S, CPCI-SSH, ESCI Timespan=All years* | [Edit](http://0-apps.webofknowledge.com.wam.leeds.ac.uk/WOS_AdvancedSearch_input.do?product=WOS&SID=E6DF347xpvyl1VP4woH&search_mode=AdvancedSearch&replaceSetId=5&editState=init) |  |  |
|  | | | | | |
| # 4 | [**1,352,463**](http://0-apps.webofknowledge.com.wam.leeds.ac.uk/summary.do?product=WOS&doc=1&qid=6&SID=E6DF347xpvyl1VP4woH&search_mode=GeneralSearch&update_back2search_link_param=yes) | **TOPIC:** (old*)  *Indexes=SCI-EXPANDED, SSCI, A&HCI, CPCI-S, CPCI-SSH, ESCI Timespan=All years* | [Edit](http://0-apps.webofknowledge.com.wam.leeds.ac.uk/WOS_AdvancedSearch_input.do?product=WOS&SID=E6DF347xpvyl1VP4woH&search_mode=AdvancedSearch&replaceSetId=4&editState=init) |  |  |
|  | | | | | |
| # 3 | [**287,873**](http://0-apps.webofknowledge.com.wam.leeds.ac.uk/summary.do?product=WOS&doc=1&qid=5&SID=E6DF347xpvyl1VP4woH&search_mode=GeneralSearch&update_back2search_link_param=yes) | **TOPIC:** (elder*)  *Indexes=SCI-EXPANDED, SSCI, A&HCI, CPCI-S, CPCI-SSH, ESCI Timespan=All years* | [Edit](http://0-apps.webofknowledge.com.wam.leeds.ac.uk/WOS_AdvancedSearch_input.do?product=WOS&SID=E6DF347xpvyl1VP4woH&search_mode=AdvancedSearch&replaceSetId=3&editState=init) |  |  |
|  | | | | | |
| # 2 | [**1,383,663**](http://0-apps.webofknowledge.com.wam.leeds.ac.uk/summary.do?product=WOS&doc=1&qid=4&SID=E6DF347xpvyl1VP4woH&search_mode=GeneralSearch&update_back2search_link_param=yes) | **TOPIC:** (Adult*)  *Indexes=SCI-EXPANDED, SSCI, A&HCI, CPCI-S, CPCI-SSH, ESCI Timespan=All years* | [Edit](http://0-apps.webofknowledge.com.wam.leeds.ac.uk/WOS_AdvancedSearch_input.do?product=WOS&SID=E6DF347xpvyl1VP4woH&search_mode=AdvancedSearch&replaceSetId=2&editState=init) |  |  |
|  | | | | | |
| # 1 | [**5,315,459**](http://0-apps.webofknowledge.com.wam.leeds.ac.uk/summary.do?product=WOS&doc=1&qid=2&SID=E6DF347xpvyl1VP4woH&search_mode=GeneralSearch&update_back2search_link_param=yes) | **TOPIC:** (Patient*)  *Indexes=SCI-EXPANDED, SSCI, A&HCI, CPCI-S, CPCI-SSH, ESCI Timespan=All years* | [Edit](http://0-apps.webofknowledge.com.wam.leeds.ac.uk/WOS_AdvancedSearch_input.do?product=WOS&SID=E6DF347xpvyl1VP4woH&search_mode=AdvancedSearch&replaceSetId=1&editState=init) |  |  |
